# High-throughput screening reveals mechanisms of environmental control of germination in a fungal thermophile

**DOI:** 10.1128/mbio.03907-25

**Published:** 2026-04-20

**Authors:** Olusola A. Ogunyewo, Kristopher Fleming, Madeleine Morris, Kayleigh Fort, Lori B. Huberman, Rachel B. Brem

**Affiliations:** 1 Department of Plant and Microbial Biology, University of California, Berkeley, California, USA; 2 Plant Pathology and Plant-Microbe Biology Section, Cornell University, School of Integrative Plant Science 5922, Ithaca, New York, USA; Instituto Carlos Chagas, Curitiba, Brazil

**Keywords:** BarSeq/TnSeq, high-throughput screening, temperature response, *Thermothelomyces thermophilus*, germination, insertional mutagenesis, zinc signaling

## Abstract

*Thermothelomyces thermophilus* is a filamentous fungus isolated from self-heating compost. Unlike most of the fungal kingdom, this species exhibits a growth optimum at 45°C and is intolerant of temperatures below 30°C. To investigate genetic contributors to temperature-dependent fitness in this system, we implemented a large-scale insertional mutagenesis approach. We generated thousands of *T. thermophilus* mutants and cultured them at temperature extremes in a standard medium. Phenotyping-by-sequencing identified dozens of disrupted loci representing candidate determinants of thermophilic life history, including several annotated in metal transport. We then validated a subset of screen hits with a directed, single-gene knockout paradigm. The results revealed a temperature-dependent regulatory logic for germination, the developmental decision by which a fungal spore initiates growth. Surprisingly, most mutants germinated far better at 50°C than the wild type in a standard medium and showed markedly slower germination at lower temperatures, consistent with altered germination regulation rather than enhanced intrinsic heat tolerance. We hypothesized that *T. thermophilus* has evolved sophisticated regulatory machinery to block germination at high temperatures unless environmental conditions are favorable. As a proof of concept, we surveyed media conditions and established that elevated zinc dampened germination of wild-type *T. thermophilus* at 50°C but promoted it at lower temperatures; mutation experiments made clear that such sensitivity was mediated in part by the zinc transporter *zip*. We interpret these results under a model in which *T. thermophilus* integrates temperature and nutrient availability to control the transition from spore dormancy to vegetative growth, a developmental decision that shapes fitness outcomes across temperatures.

**IMPORTANCE:**

Fungal thermophiles thrive at temperatures that represent the upper limits of eukaryotic life. The regulatory and developmental mechanisms that shape their temperature-dependent fitness remain poorly understood. In this work, we elucidate how *Thermothelomyces thermophilus* integrates temperature cues with other environmental inputs during germination, a key life-cycle stage for dispersal. Our findings highlight germination regulation as an important contributor to fitness at elevated temperatures in a thermophilic eukaryote. These insights are of basic biological interest and provide a foundation for rational strategies to modulate temperature-dependent performance in industrial strains, with applications for high-temperature bioprocessing.

## INTRODUCTION

Thermophilic fungi operate at the upper temperature range of eukaryotic life, with a physiological ceiling approaching ~60°C (1). Microbial ecology surveys have outlined the narrow diversity of truly thermophilic fungal species adapted to composts and geothermal soils (2, 3). In thermophilic bacteria, elevated growth temperatures have been linked to genome streamlining, higher GC content, thermostable protein folds, membrane lipid saturation, and enhanced chaperone and DNA-repair capacities (4, 5). Whether similar mechanisms govern thermophily in fungi remains unclear, particularly given the added complexity of organelles, chromatin regulation, and developmental programs in these systems. Thus, against the backdrop of years of work on mesophile and thermotolerant fungi (6–9), the mechanisms of high-temperature growth preference in fungal thermophiles are still incompletely understood.

Beyond their fundamental biological interest, thermophilic fungi also have practical relevance as potential hosts for industrial bioprocessing. Microbial cell factories are widely engineered to produce fuels, pharmaceuticals, and other industrially valuable chemicals; however, the cost of cultivation substrates remains a major constraint to sustainable biomanufacturing (10, 11). Fungi represent a potential solution toward this end, when they have the capacity to digest abundant, low-cost crop wastes like rice straw and corn stover. For such substrates, industrial processes commonly apply high-temperature pretreatments to accelerate biomass deconstruction. In this pipeline, subsequent cooling prior to microbial inoculation adds cost and energy demand (12). Thermophilic fungi therefore represent a promising chassis for bioproduction, as they could bridge pretreatment and fermentation steps without the need for cooling, directly converting pretreated biomass into target products.

Among thermophilic fungi, *Thermothelomyces thermophilus* (formerly *Myceliophthora thermophila*) has emerged as a compelling model with robust growth near 50°C and a carbohydrate-active enzyme repertoire capable of plant cell-wall deconstruction, sparking interest in its use as a thermophilic cell factory (13, 14). Genetic tools in this system now enable precise engineering (15), including the potential to optimize high-temperature performance (16–18). We set out to use *T. thermophilus* as a testbed for the dissection of mechanisms of fungal thermophily at a genomic scale. Our approach was to pioneer the application of a recently developed method for randomly barcoded insertional mutagenesis with screening via sequencing in filamentous fungi (19) for the *T. thermophilus* system. We sought to use this strategy for an unbiased genetic survey of temperature-responsive growth behaviors in *T. thermophilus*. We used the results as a jumping-off point for exploration of the temperature dependence of the asexual stages of the filamentous fungal life cycle and the underlying genetics. The results reveal how a thermophilic eukaryote coordinates stress signaling and development at high temperature.

## RESULTS

### Temperature-dependent growth and development of *T. thermophilus*

**Fig 1:**
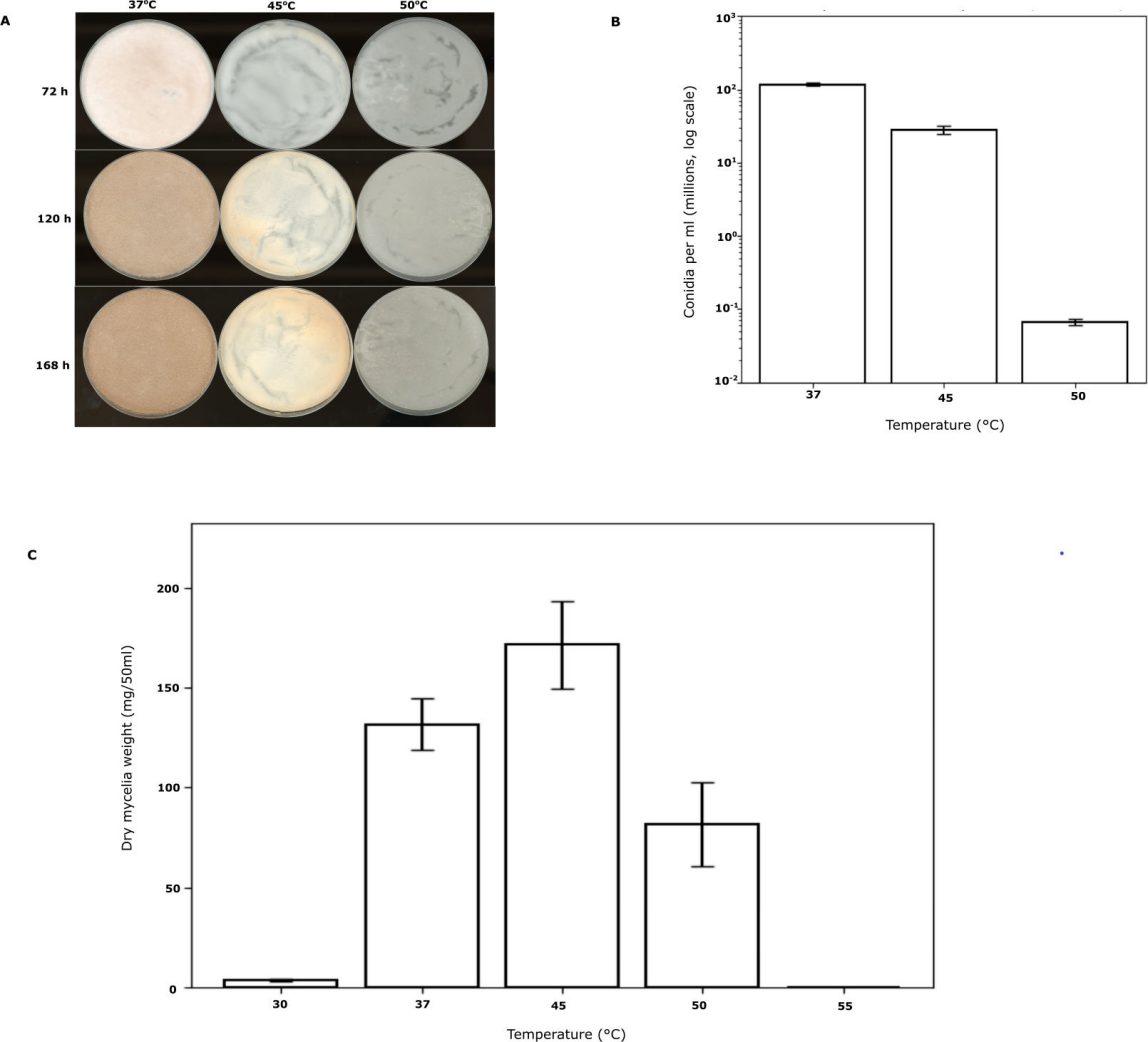
Growth and developmental measurements of wild-type *T. thermophilus* across temperatures. (**A**) Colony morphology on solid Vogel’s medium at 37°C, 45°C, and 50°C was imaged at 72, 120, and 168 h post-inoculation. (**B**) Conidial density was measured from cultures grown at 37°C, 45°C, and 50°C after 168 h. The *x*-axis indicates growth temperature (°C), and the *y*-axis indicates conidia per milliliter (log scale). Bars represent mean ± SD from three biological replicates. (**C**) Dry mycelial biomass was measured from 50 mL liquid cultures incubated for 24 h at the indicated temperatures. The *x*-axis indicates growth temperature (°C), and the *y*-axis indicates dry mycelial weight (mg per 50 mL culture). Bars represent mean ± SD from three biological replicates.

With the ultimate goal of understanding temperature-dependent growth and developmental behaviors in *T. thermophilus*, we first surveyed growth behaviors of *T. thermophilus* across temperatures and conditions. On solid agar, we documented the asexual growth cycle, from hyphal expansion through the formation of aerial hyphae to the production of spores (conidia) with visible pigmentation (Fig. 1A). We observed a temperature-responsive lag in development as the temperature increased, even with high-percentage agar and high-humidity incubation to maintain plate integrity (Fig. 1A and B). As a complement, we also assayed growth in liquid culture, in which we inoculated *T. thermophilus* conidia into standard liquid medium and quantified the resulting mycelial biomass accumulation after incubation with shaking. The results revealed evidence for *bona fide* thermophilicity by *T. thermophilus*, with an optimum at 45°C during liquid growth and a gradual falloff of performance under warmer and cooler conditions (Fig. 1C). We thus considered liquid culture as a growth format suitable for genetic analyses to reveal mechanisms contributing to temperature-dependent fitness in *T. thermophilus*.

### Construction and mapping of a *T. thermophilus* insertional mutant library

**Fig 2:**
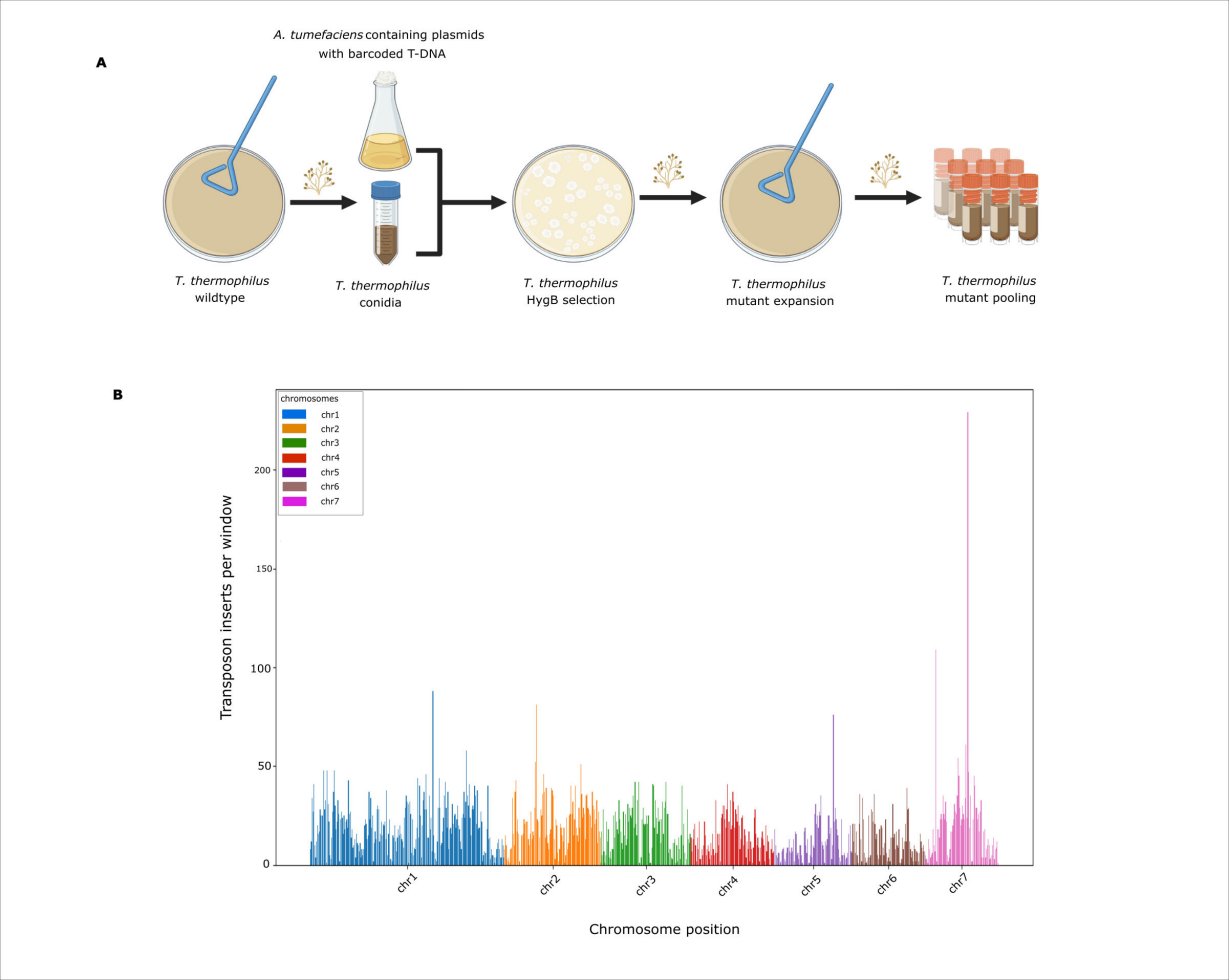
Construction and genomic distribution of a *T. thermophilus* barcoded insertional mutant pool. (**A**) Overview of the *Agrobacterium*-mediated transformation workflow used to generate the barcoded insertion mutant pool. *T. thermophilus* conidia were co-cultivated with *A. tumefaciens* containing plasmids with barcoded T-DNA. Transformants were selected on hygromycin-containing medium. Transformants were expanded by allowing them to conidiate on selection plates, and conidia from all plates were harvested and bulked to generate the barcoded insertion mutant pool. (**B**) Genome-wide mapping of T-DNA insertions across the seven chromosomes of *T. thermophilus*. The *x*-axis indicates chromosomal position (grouped by chromosome), and the *y*-axis indicates the number of mapped T-DNA insertions per 50 kb genomic window. Colors correspond to individual chromosomes.

To develop a genomic resource for mapping genotype to phenotype in *T. thermophilus*, we constructed a pool of barcoded insertional mutants. Our process used random insertion from *Agrobacterium tumefaciens* harboring hygromycin-marked, barcoded transfer DNA (T-DNA) (19), with which we carried out ectopic transformation of mononucleate *T. thermophilus* conidia via established procedures (20) with minor modifications to conidial density, co-cultivation duration, and selection conditions, followed by expansion and aliquoting of replicate sub-pools (Fig. 2A). To catalog the diversity and chromosomal distribution of T-DNA insertions across the mutants, we isolated DNA from replicate cultures of the pool grown in liquid medium at 37°C and prepared sequencing libraries enriched for T-DNA–genome junctions. *In silico* analysis of reads mapped to the *T. thermophilus* reference genome identified the T-DNA right-arm junction, extracted adjacent genomic sequence, and assigned uniquely mapped barcodes to genomic coordinates. From these data, we detected 17,155 distinct insertion sites across all seven chromosomes (File S1). Their distribution was largely uniform with a few prominent peaks (Fig. 2B); most notable of the latter was a T-DNA insertion hotspot on chromosome 7 corresponding to an rRNA-encoding locus (File S2), suggestive of high transcriptional activity as a driver of insertion prevalence. In total, 3,426 T-DNA insertions were located within annotated genes (File S1), establishing our pool as a partial-coverage mutant collection suitable for quantitative fitness profiling in *T. thermophilus*.

### Genome-wide quantification of temperature-dependent mutant fitness

**Fig 3:**
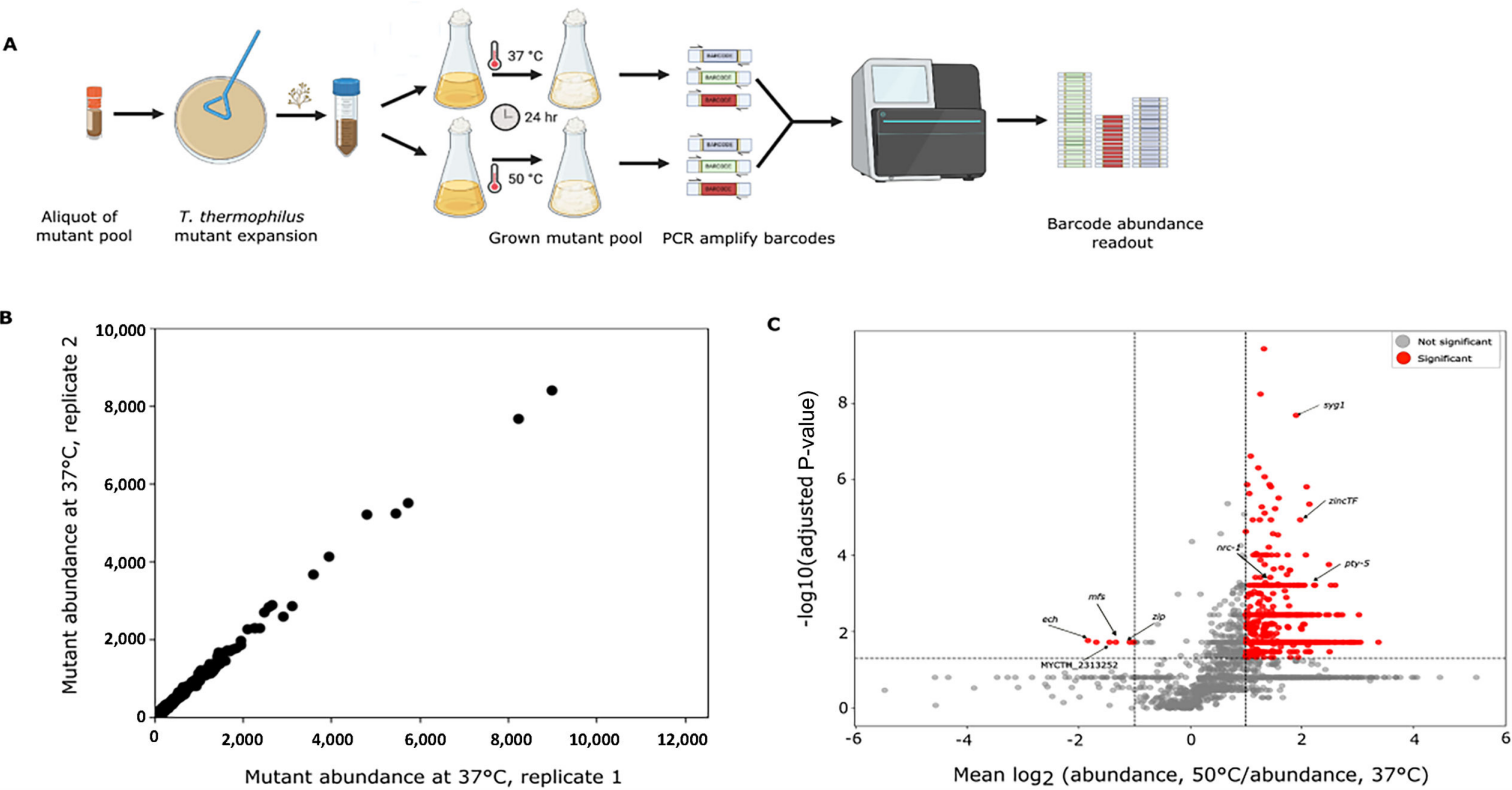
Genome-wide barcode sequencing (BarSeq) fitness profiling in *T. thermophilus* identifies temperature-dependent mutant defects. (**A**) Experimental workflow for BarSeq of the *T. thermophilus* mutant pool. Conidia were inoculated from the pool stock onto solid medium, harvested, and used to start liquid cultures. Cultures were incubated for 24 h at 37°C or 50°C. Genomic DNA was extracted from each culture, and barcode regions were amplified and sequenced. Sequencing reads were used to quantify barcode abundance for downstream fitness analysis. (**B**) Example comparison of mutant abundances between two biological replicates grown at 37°C. Each point represents one mutant, and the *x*- and *y*-axes report normalized abundance in the indicated replicate. (**C**) Each point reports the results of a test for the differential abundance of insertional mutants in one gene between temperatures. The *x*-axis reports the log of the ratio of two quantities: the average abundance, across genotypes and biological replicates, of mutants at the respective gene at 50°C, and the analogous quantity at 37°C. The *y*-axis indicates –log₁₀(adjusted *P*-value) from statistical testing for differential abundance of the respective mutants between temperatures. Points in red indicate loci meeting quality control thresholds for temperature-dependent abundance change in terms of significance, effect size, and variability (see Materials and Methods). Genes chosen for single-gene follow-up are labeled with arrows.

We expected that screens with our insertional mutant pool could enable the dissection of temperature-dependent phenotypes in *T. thermophilus*. To this end, we sought to use the resource to identify genes whose mutation effect on fitness depended on temperature. We designed an experimental scheme using growth assays of our mutant pool at 37°C and 50°C, representing temperatures that approached the lower and upper extremes, respectively, of wild-type temperature tolerance in liquid culture (Fig. 1C). For each biological replicate, we cultured independent aliquots of the *T. thermophilus* mutant pool in parallel at 37°C and 50°C, and we quantified mutant abundance by sequencing the barcode regions of the inserted T-DNA from the resulting genomic DNA (Fig. 3A). In the resulting abundance data, reproducibility between biological replicates was high across both temperatures (Pearson r > 0.994; Fig. 3B; Fig. S1). We used the complete data set as input into a quality control and analysis pipeline (see Methods) that assessed differences between temperatures in mutant abundance on a per-gene basis, from which 783 mutants emerged at adjusted *P*-value < 0.05 and absolute log_2_(fold change) ≥ 1 (File S3). Although the genes in which these mutations lay spanned a range of annotated functions (File S3), their behavior in the screen was remarkably consistent: for all but six gene hits, mutant abundance was higher at 50°C than at 37°C (Fig. 3C; File S3). These data attested to a highly complex genetic architecture of temperature response in *T. thermophilus,* whose contributors, when mutated, almost invariably conferred defects in cooler conditions and/or benefits at high temperature.

### Single-gene validation of temperature-dependent screen hits

To validate the inferences from our genome-wide random mutagenesis screen, we took a single-gene approach. For this purpose, we chose eight screen hits (Fig. 3C; Table 1) with high coverage in the mutant pool and robust mutant effects favoring either 50°C or 37°C. These included putative regulators (*zincTF, syg1*, *pty-5*, and *nrc-1*), transporters (*zip* and *mfs*), and housekeeping genes (*ech* and *MYCTH_2313252*) (Table 1). We generated targeted deletion mutants for each by homologous recombination of a hygromycin‐resistance cassette at the endogenous *T. thermophilus* locus, in a strain harboring a deletion in *ku80* to ensure efficient deletion by eliminating non-homologous end joining. Correct integration events were confirmed by PCR and sequencing (Fig. S2).

**TABLE 1:** Hits from high-throughput screen subjected to single-gene validation^*a*^

GeneID	Gene symbol	Protein name	Mutant effect on abundance, log_2_(50°C/37°C)	Adjusted *P*-value
MYCTH_2139072	*zincTF*	Zn(2)-C6 fungal-type domain-containing protein	1.98	1.12 E−05
MYCTH_2300403	*pty-5*	Tyrosine-specific protein phosphatase-domain-containing protein	1.95	5.84 E−02
MYCTH_100809	*syg1*	SPX domain-containing protein	1.90	2.01E−08
MYCTH_2305107	*nrc-1*	MAPKK kinase	1.15	8.6 E−04
MYCTH_116881	*mfs*	MFS general substrate transporter	−0.98	0.0184
MYCTH_2303755	*ech*	Enoyl-CoA hydratase	−1.40	0.0078
MYCTH_2307966	*zip*	Zinc/iron permease	−1.45	0.0184
MYCTH_2313252	MYCTH_2313252	Putative UPF0658 Golgi apparatus membrane protein	−1.83	0.0171

^*a*^ Each row reports effects of mutation of one gene on temperature-dependent fitness as measured by large-scale pooled T-DNA mutagenesis, from Fig. 3C, for a locus chosen for follow-up single-gene validation analysis. For each gene, the table reports the *T. thermophilus* gene identifier (GeneID); the corresponding gene symbol; the annotated protein name; the log of the ratio of two quantities: the average abundance, across genotypes and biological replicates, of mutants at the respective gene at 50°C, and the analogous quantity at 37°C; and the adjusted *P*-value from a Wilcoxon rank-sum test for differential abundance of the respective mutants between temperatures.

**Fig 4:**
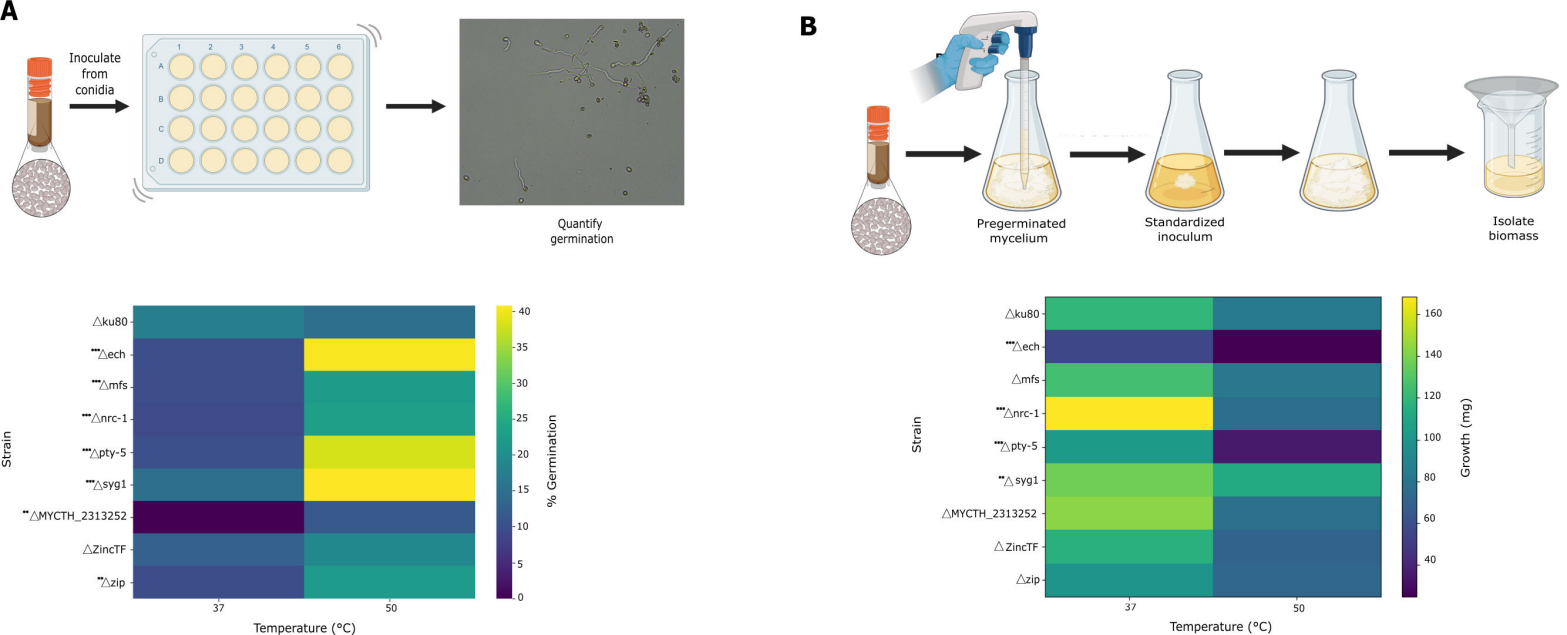
Germination and biomass measurements for single-gene deletion strains of *T. thermophilus*. (**A**) Top, germination assay. Conidia were inoculated into microtiter wells and incubated at a temperature of interest, followed by scoring via microscopy. Bottom, each row reports results from germination assays of the indicated strain at the indicated temperatures. Each cell reports the average, across replicates, of the percent of conidia scored as germinating. (**B**) Top, biomass assay. Conidia were inoculated into liquid medium and incubated to allow germination and pre-growth; the resulting mycelium was used as inoculum into a second culture, followed by incubation at a temperature of interest, and biomass harvest and quantification. Bottom, each row reports results from growth assays of the indicated *T. thermophilus* strain at the indicated temperatures. Each cell reports the average, across replicates, of dry biomass (mg) for each strain at each temperature. Statistical significance was evaluated using a two-way ANOVA (strain × temperature) with *post-hoc* comparisons relative to the Δku80 reference strain. Asterisks denote significance (*P < 0.05; **P < 0.01; ***P < 0.001).

In designing experiments to characterize these single-gene mutant strains, we reasoned that temperature could influence multiple stages of the *T. thermophilus* life cycle, such that distinguishing between such effects would maximize our insights into the mechanisms of temperature response. We elected to focus on the phases relevant for the growth scheme of the high-throughput screen (Fig. 3), namely germination of conidia and the extension of hyphae after vegetative growth had established. To this end, we established an assay to quantify temperature impacts on conidial germination of a given strain by microscopy, at a relatively early time point where germlings could easily be resolved from one another in an imaging field (Fig. 4A). Separately, we designed an assay of temperature-dependent vegetative hyphal extension for a given strain, avoiding confounding effects from germination by using a standardized pre-germinated inoculum (Fig. 4B). We applied both assays to each mutant and to their isogenic parent, the otherwise wild-type Δ*ku*80 strain. This scheme allowed us to distinguish effects on germination from effects on hyphal growth once a colony was established, in the interpretation of mutant phenotypes.

From germination measurements across this strain set, we observed a modest drop in performance at elevated temperature by the Δ*ku80* parent strain, which we used as a baseline for comparison of the remainder of the single-gene mutants (Fig. 4A). Among these strains, six of eight genotypes exhibited a marked advantage at 50°C, germinating up to 2-fold more avidly than the parent; by contrast, at 37°C, six of eight mutants exhibited reduced germination relative to the parent (Fig. 4A). In these validated cases, mutations at a given locus (*ech, mfs, nrc-1, pty-5, syg1,* and *zip*) most often exerted effects in opposite directions at the two temperatures (Fig. 4A), attesting to dual roles of a given gene with respect to germination at the two extremes. Their trends mirrored the results from our large-scale screen, in which fitness phenotypes of mutants made by random integration almost universally favored high abundance at 50°C (Fig. 3B). Interestingly, in three exceptions to the latter (*mfs*, *ech,* and *zip*), for which mutant abundance in the screen was highest at 37°C (Fig. 3B; Table 1), our single-gene validation experiments nonetheless established that the respective mutants germinated preferentially at 50°C. This observation, and the fact that a single-gene deletion of *MYCTH_2313252* conferred a germination defect at the lower temperature (Fig. 4A) but random insertional mutants at this locus in the conditions of the screen were highly abundant at 37°C relative to 50°C (Fig. 3B; Table 1), suggested cases in which performance in the growth scheme of the screen reflected contributions from behaviors other than those interrogated in our germination assay. However, apart from Δ*MYCTH_2313252*, the most salient and pervasive pattern from our observations of germination in single-gene mutants was that of improvements at high temperatures (Fig. 4A). We thus formulated a model in which the wild-type alleles of many loci function as repressors of germination at high temperatures, such that their deletion would release this brake and allow development to proceed despite heat stress; the same loci would act as activators of germination under cooler conditions.

We next turned to the assays of biomass accumulation in the hyphal growth phase of *T. thermophilus*, inoculating with pre-germinated mycelium at an equal starting concentration for all strains, including the Δ*ku80* parent (see Materials and Methods). For wild-type *T. thermophilus*, high temperature retarded growth in this scenario, with 1.5-fold lower biomass accumulation at 50°C than at 37°C (Fig. 4B). Most single-gene mutants phenocopied this behavior almost exactly, such that we identified few cases in which a mutant grew differently from the wild-type parent at either temperature (Fig. 4B). Among these exceptions, we noted an advantage of the *nrc-1* deletion mutant relative to wild-type at 37°C; defects at 50°C in the *Δpty-5* and *Δech* strains (representing a plausible mechanism for increased abundance of mutants at these loci at 37°C in our original large-scale screen [Fig. 3B; Table 1]); and increased growth by *Δsyg-1* at 50°C (Fig. 4B). However, as the latter represented outliers across the gene set, we concluded that growth was not a primary life cycle stage for the roles of most of our focal genes in temperature response, particularly in light of the striking impact of their deletions during germination (Fig. 4A). Given the origin of our focal genes as representative of the hundreds of hits from our large-scale screen (Fig. 3B), we infer that the overall trends in our screen results were also likely dominated by temperature-dependent effects of gene disruption on germination rather than vegetative growth.

### Zinc availability modulates germination efficiency

**Fig 5:**
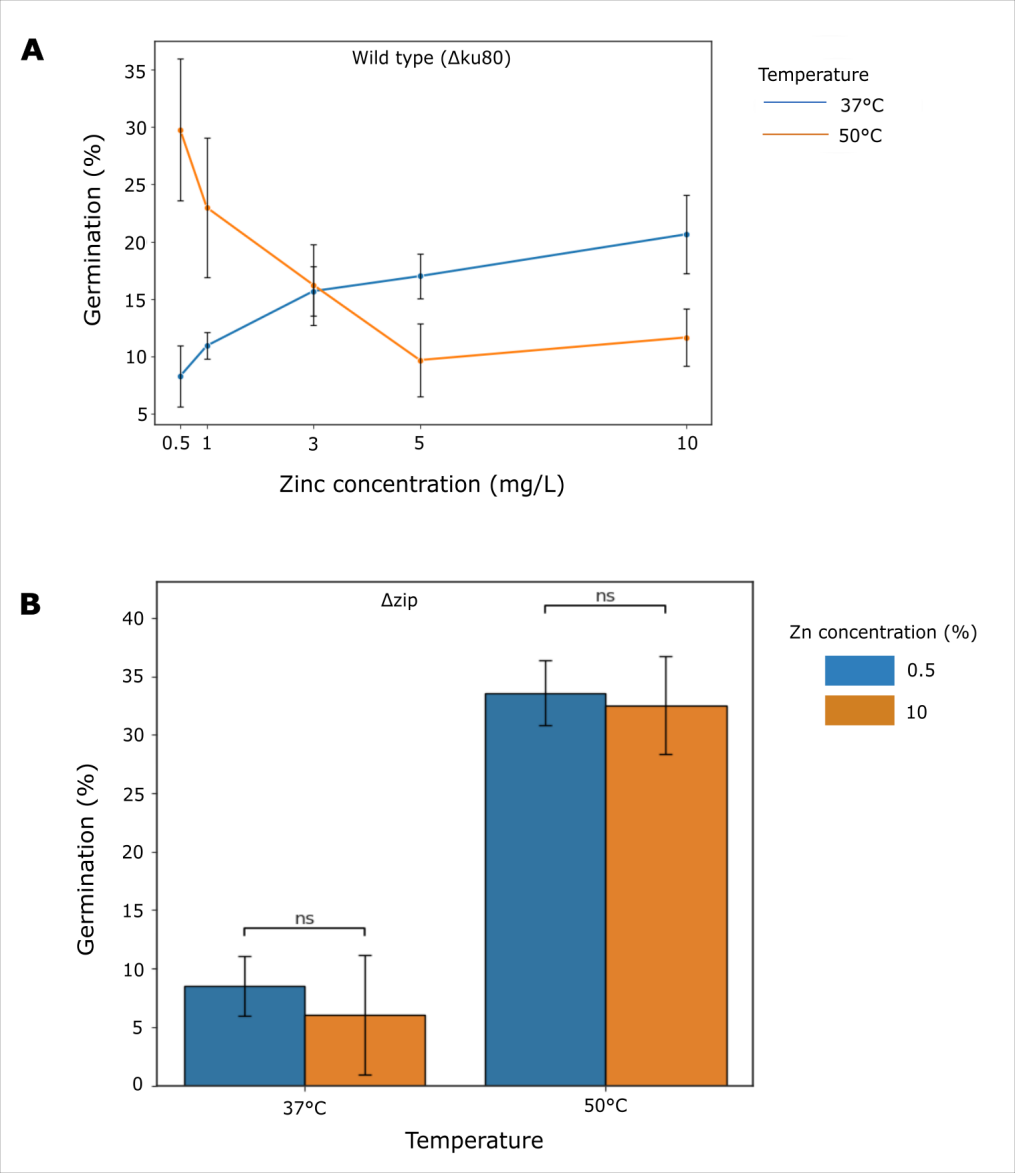
Zinc-dependent modulation of germination in *T. thermophilus*. (**A**) Germination of wild-type *T. thermophilus* across zinc concentrations and temperatures. The *x*-axis indicates zinc concentration in the medium (mg/L), and the *y*-axis indicates the percentage of germinated conidia. Each point represents the mean value, and error bars denote standard deviation across biological replicates. (**B**) Germination of *T. thermophilus* Δ*zip* across zinc concentrations and temperatures. The *x*-axis indicates temperature, and the *y*-axis is as in panel **A**. Each bar represents the mean value, and error bars denote standard deviation across biological replicates. ns, not significant in a *t*-test comparing germination between zinc concentrations for the indicated temperatures.

Is there a regulatory logic that governs repression of germination in wild-type *T. thermophilus* at high temperature? We hypothesized that the fungus integrates multiple environmental inputs, including temperature and nutrient availability, in the decision to germinate. As a test case, we considered environmental metal exposure, in light of the validated role in temperature-dependent germination of the putative zinc-iron permease *zip* (Fig. 4A). We formulated a model in which zinc availability acts to gate *T. thermophilus* germination under heat stress. To explore this, we performed a zinc titration series for wild-type *T. thermophilus*, assaying germination as a function of zinc concentration and temperature. The results revealed a dose-responsive benefit in germination from increasing zinc at 37°C and the inverse relationship at 50°C, with germination falling off markedly as zinc concentrations increased (Fig. 5A). At the lowest zinc concentrations, germination at high temperature far outstripped that observed at 37°C (Fig. 5A). Such a pattern dovetailed with the benefit to germination at 50°C that we had observed in standard medium (5 mg/L zinc) upon deletion of *zip* (Fig. 4A), as expected if zinc uptake were limited in the Δ*zip* mutant. To test the role of *zip* directly, we repeated germination assays in the Δ*zip* strain and found no impact of zinc concentration on germination efficiency in this background (Fig. 5B). Rather, germination remained higher at 50°C than at 37°C across zinc conditions in the absence of *zip* (Fig. 5B). Together, these data demonstrate that zinc is a key environmental input into developmental decision-making in *T. thermophilus* and that zinc-dependent modulation of germination requires *zip*.

## DISCUSSION

Thermophilic fungi are a powerful model for the basic biology of how eukaryotes adapt to extreme environments, and they are also of interest in industrial applications. In this work, we have established insertional mutagenesis as a tool for the mechanistic dissection of temperature response in *T. thermophilus*. High-throughput forward genetics in filamentous fungi is in its infancy (19), and our method is well-positioned to accelerate advances in the *T. thermophilus* system, as has now become standard in bacteria and yeasts (21–25). The principles that have emerged from our study break new ground in the understanding of the thermophilic life history for fungi.

We have shown that hundreds of genes, when disrupted in our high-throughput screening approach, affect high-temperature performance by *T. thermophilus*. Among these, our validation of focal gene hits has found that most act as negative regulators of conidial germination at high temperature, in that they improve germination when deleted. The capacity to make spores represents a mechanism for stress resistance common to essentially all fungi, and exit from this dormant state is tightly regulated even in mesophiles (26–28) to ensure that a new colony only commits to growth under favorable conditions. It is tempting to speculate that for thermophiles, evolution has optimized the dormancy-breaking decision in unique ways, taking into account the cost or toxicity of growth programs at extreme temperatures. If so, the logic in *T. thermophilus* would be to sense environmental signals associated with the worst risk at high temperature and use them to trigger a block on germination.

Our screen and validation data raise the possibility that zinc processing represents one such environmental risk factor. If cellular functions involving zinc were particularly deleterious during *T. thermophilus* germination under high-heat conditions, it would mirror previous studies in mesophilic fungi in which metal exposure compromises spore germination more than it does hyphal growth (29). Plausibly, such effects could be mediated by reactive oxygen species, which have been associated with both zinc (30, 31) and elevated temperature (32, 33) in other systems. Formally, however, our data leave open the question of whether and how zinc exerts damaging effects *per se* in *T. thermophilus*. What our experiments have shown is that levels of zinc that shut down germination at high temperatures fostered the best germination at 37°C. This pattern is consistent with a regulated delay in germination under combined zinc and heat stress, rather than solely a passive consequence of zinc-induced damage; indeed, the zinc concentrations we have tested here fall within ranges commonly used in fungal physiology studies (34–36). That said, we see zinc as only one example of an environmental cue that can be integrated with thermal signals to shape developmental decisions by *T. thermophilus*. We expect that the annotated regulator genes whose deletion boosts germination at high temperature will ultimately prove to represent nodes of a complex signal-integration network controlling the *T. thermophilus* life cycle. Such a function could reflect repurposing from the known roles, in mesophiles, of our focal genes *pty-5* (a tyrosine phosphatase) and *nrc-1* (a MAP3 kinase) in conidiation and germling fusion, respectively (37, 38); the function of another of our focal factors, *syg-1,* likely in phosphate-based signaling (39), is unknown even in model fungi. For the annotated effector genes we have highlighted here—*ech* (encoding enoyl-CoA hydratase, a β-oxidation enzyme whose disruption alters growth and development in other fungi) and the zinc/iron permease gene *zip* (40, 41)—we interpret their mutant effects as eliminating repressive environmental inputs farther upstream, allowing germination to proceed even under heat stress.

Looking ahead, although we have emphasized relief of germination repression in *T. thermophilus* mutants, our work has also uncovered genes for which we infer positive effects on mycelial growth (focal genes *ech* and *pty-5*); that is, their deletion compromises this phase of the life cycle at high temperatures. We anticipate that many more such pro-growth factors remain to be identified in *T. thermophilus*. It is reasonable to assume that principles from thermophilic bacteria and archaea that lack multiple life stages, in which evolution has adjusted housekeeping genes all over the genome to promote growth at high temperature (42), are likely to bear out in fungi. Our screening approach for *T. thermophilus* may have been particularly likely to miss pro-growth loci of this kind if they are essential, in that their disruption mutants would fail to propagate in our stocks at any temperature and would be lost from our analysis pipeline. As such, and in light of the partial coverage of our mutant pool, our screen results represent only a first window onto the complete suite of genes that mediate the thermophilic life history of *T. thermophilus*. As they stand, however, our findings underscore the importance of germination as a nexus of regulatory decisions for *T. thermophilus*, integrating temperature with other environmental inputs—which could well include nutrient availability, pH, or osmotic or oxidative stress, as well as metal homeostasis—to optimize fitness.

### Conclusion

We have used an insertional mutagenesis screen and validation follow-up in *T. thermophilus* to uncover principles of thermophilicity in fungi. Our results shed light for the first time on the regulatory landscape of decisions by these organisms about whether to germinate in the heat. In an industrial setting, the deletion mutants we study here represent compelling candidate cases in which genetic manipulations could improve performance by *T. thermophilus* even at the extreme of its heat tolerance.

## MATERIALS AND METHODS

### Strains, media, and growth conditions

*T. thermophilus* ATCC 42464 was obtained from the American Type Culture Collection. The ∆*ku80* strain MJK20.2 was generously provided by Vera Meyer (18, 43). The strains were maintained on Vogel’s minimal medium (VMM) (44) supplemented with 2% sucrose. For solid cultures, VMM with the indicated carbon source was solidified with 3% (wt/vol) agar. Liquid cultures were grown in 250 mL Erlenmeyer flasks at 200 rpm with 50 mL culture volume. Unless otherwise stated, cultures were incubated at 37°C, 45°C, or 50°C in a closed, dark incubator using the New Brunswick Innova 40 incubator. Conidia were harvested from 7-day-old plates by gentle flooding with sterile 0.01% (vol/vol) Tween-80, filtered through sterile Miracloth to remove hyphal fragments, counted using a hemocytometer, and used immediately or stored at −80°C until use. All other strains and plasmids generated from the study are listed in Table 2.

**TABLE 2:** List of strains and plasmids used for this study

Strain or plasmid	Description	Reference or source^*a*^
Strains		
*Escherichia coli* DH5α	F− Φ80lacZΔM15 Δ(lacZYA-argF) U169 recA1 endA1 hsdR17	Invitrogen
*Agrobacterium tumefaciens* LBA4404	Ach5 with pAch5, Δ*tra* Δ*occ* ΔT_L_ ΔT_R_ Rif^r^	(45)
*Thermothelomyces thermophilus* ATCC 42464	Wild-type reference strain used as the parental background in this study	
MJK20.2 (∆*ku*80)	Mutant strain of *T. thermophilus* having deletion of *ku*80 gene (MYCTH_2118116)	
∆*ech*	Mutant strain of *T. thermophilus* having deletion of *ech* (MYCTH_2303755) and *ku*80 (MYCTH_2118116) genes	This study
∆*mfs*	Mutant strain of *T. thermophilus* having deletion of *mfs* (MYCTH_116881) and *ku*80 (MYCTH_2118116) genes	This study
∆*nrc-1*	Mutant strain of *T. thermophilus* having deletion of *nrc-1* (MYCTH_2305107) and *ku*80 (MYCTH_2118116) genes	This study
∆*pty-5*	Mutant strain of *T. thermophilus* having deletion of *pty-5* (MYCTH_2300403) and *ku*80 (MYCTH_2118116) genes	This study
∆*syg1*	Mutant strain of *T. thermophilus* having deletion of *syg1* (MYCTH_100809) and *ku*80 (MYCTH_2118116) genes	This study
∆*MYCTH_2313252*	Mutant strain of *T. thermophilus* having deletion of *MYCTH_2313252* and *ku*80 (MYCTH_2118116) genes	This study
∆*zincTF*	Mutant strain of *T. thermophilus* having deletion of *zincTF* (MYCTH_2139072) and *ku*80 (MYCTH_2118116) genes	This study
∆*zip*	Mutant strain of *T. thermophilus* having deletion of *zip* (∆MYCTH_2307966) and *ku*80 (MYCTH_2118116) genes	This study
Plasmids		
pCambia 1302	Kanamycin- and hygromycin-resistant vector containing EGFP	(46)
pLH159RB	Plasmids for *A. tumefaciens*-mediated transformation with barcoded T-DNA	(19)
pOORB007	pCAMBIA1302-based plasmid bearing the *nrc-1* deletion cassette, consisting of ~1 kb upstream and downstream homology arms flanking the hph selectable marker in place of the *nrc-1* ORF	This study
pOORB008	pCAMBIA1302-based plasmid bearing the *syg1* deletion cassette, consisting of ~1 kb upstream and downstream homology arms flanking the hph selectable marker in place of the *syg1* ORF	This study
pOORB009	pCAMBIA1302-based plasmid bearing the *ech* deletion cassette, consisting of ~1 kb upstream and downstream homology arms flanking the hph selectable marker in place of the *ech* ORF	This study
pOORB012	pCAMBIA1302-based plasmid bearing the *zincTF* deletion cassette, consisting of ~1 kb upstream and downstream homology arms flanking the hph selectable marker in place of the *zincTF* ORF	This study
pOORB014	pCAMBIA1302-based plasmid bearing the *pty-5* deletion cassette, consisting of ~1 kb upstream and downstream homology arms flanking the hph selectable marker in place of the *pty-5* ORF	This study
pOORB018	pCAMBIA1302-based plasmid bearing the *mfs* deletion cassette, consisting of ~1 kb upstream and downstream homology arms flanking the hph selectable marker in place of the *mfs* ORF	This study
pOORB020	pCAMBIA1302-based plasmid bearing the MYCTH_2313252 deletion cassette, consisting of ~1 kb upstream and downstream homology arms flanking the hph selectable marker in place of the MYCTH_2313252 ORF	This study
pOORB021	pCAMBIA1302-based plasmid bearing the *zip* deletion cassette, consisting of ~1 kb upstream and downstream homology arms flanking the hph selectable marker in place of the *zip* ORF	This study

^*a*^ Reference or source for strain construction and plasmid generation.

### Temperature-dependent growth and development assays of wild-type *T. thermophilus*

To characterize thermal performance, Conidia were inoculated onto solid VMM + 2% sucrose plates at a final density of 1.2 × 10^6^ conidia/spot and incubated at 37°C, 45°C, or 50°C for 168 h in a closed incubator under standard dark conditions. Growth was observed daily using a flatbed scanner. Colony pigmentation and aerial hyphae formation were documented under standardized lighting conditions. Conidiation output was quantified from cultures grown on solid medium for 168 h. Conidia were harvested as described above and counted using a hemocytometer in triplicate biological replicates per temperature. For liquid growth assays, conidia were inoculated at 1 × 10⁶ conidia/mL into 50 mL VMM + 2% sucrose in 250 mL Erlenmeyer flasks and incubated at 30°C, 37°C, 45°C, 50°C, or 55°C for 24 h at 200 rpm under standard dark conditions. Biomass accumulation was quantified by dry weight after filtration onto pre-weighed Whatman GF/A filters and dried at 80°C until a constant mass was achieved.

### Construction and tnseq of the insertional mutant pool

We generated genome-wide random insertional mutant libraries in *T. thermophilus* ATCC 42464 using *A. tumefaciens*-mediated transformation. To obtain fresh starting material, we prepared conidia on VMM containing 2% sucrose and 2% agar and incubated the plates at 37°C for 5–7 days in the dark. Conidia were harvested in sterile water, filtered through Miracloth to remove hyphal debris, counted with a hemocytometer, and adjusted to ~4 × 10^7^ conidia/mL. For *Agrobacteriu*m transformation, we used *the A. tumefaciens* strain EHA105, which carries a binary vector encoding a barcoded T-DNA cassette and a hygromycin resistance marker (19). The strain was cultured in low-salt LB with kanamycin in an exponential phase (OD_600_ ~0.5–0.8), then transferred to the induction medium (IM) containing 19.5 g/L 2-(N-morpholino)ethane sulfonic acid (MES) at pH 5.5 supplemented with 500 μM acetosyringone for 18 h to induce virulence following the modified method in reference 47. Grown *A. tumefaciens* (OD_600_ ~0.3–0.7) was mixed with *T. thermophilus* conidia at a 1:1.5 ratio and co-incubated for 1 h at room temperature with gentle mixing to prevent settling.

We spread 1 mL of the co-culture mixture onto the surface of a sterile ultra-clear cellophane (#RPI-1090) placed on a 150 × 15 mm IM agar plate supplemented with 500 μM acetosyringone. Plates were incubated at 28°C for 96 h before transferring the cellophane overlays to VMM + 2% sucrose selection plates containing 200 μg/mL hygromycin and 100 μg/mL carbenicillin. Plates were incubated at 37°C until hygromycin-resistant colonies emerged (4–7 days), and conidiation was allowed to proceed for an additional ~14 days. This full workflow was performed twice, generating two independent mutant pools: Batch 1 (~4,000 transformants) and Batch 2 (~13,000 transformants). For each batch, conidia from all selection plates were harvested, pooled, filtered through Miracloth, and stored as 10% glycerol stocks at –80°C. Together, these two batches comprised ~20,000 unique insertion mutants.

For mapping T-DNA insertion sites (Tnseq), for each batch, we inoculated pooled mutant conidia (1 × 10⁶ conidia/mL) into 100 mL VMM + 2% sucrose and grew the culture for 24 h at 37°C and 200 rpm in the dark before extracting genomic DNA using the Quick-DNA Fungal/Bacterial Miniprep Kit (Zymo Research). No dropout mix was used at any stage, as *T. thermophilus* is not maintained as an auxotrophic system; transformants were selected solely on hygromycin-containing medium, and only disruptions in essential genes would be expected to drop out of the pooled library. We verified DNA integrity by agarose gel electrophoresis and quantified it with a Qubit dsDNA HS assay. We prepared T-DNA genome junction sequencing libraries following the general TnSeq workflow of reference 2, but using an enzymatic-fragmentation workflow based on the NEBNext Ultra II FS DNA Library Prep Kit with Sample Purification Beads for Illumina (New England Biolabs #E6177S). Library preparation followed the manufacturer’s instructions with modifications appropriate for TnSeq-style T-DNA junction enrichment as in reference 48. Briefly, FS-based fragmentation and end-repair, adaptor ligation, and dual-SPRI size selection were performed using components of the NEBNext kit, after which nested PCR enrichment of junction fragments was carried out using the Nspacer_barseq_universal and P7_MOD_TS_index primers (sequences in File S4). Final libraries were sequenced as paired-end 150 bp reads on an Illumina MiSeq at the UC Berkeley Vincent J. Coates Genomics Sequencing Laboratory.

### Tnseq analysis/mapping of the insertional mutant pool

To determine the genomic coordinates of each barcoded insertion, sequencing reads from Tnseq from Batch 1 and Batch 2 (see above) were combined and then processed using a modified version of the pipeline in reference 49. All custom modifications and scripts used in this study are available in the accompanying GitHub repository (https://github.com/oaogunyewo-git/Thermothelomyces-Tnseq-project/tree/main/TnSeq_BarSeq_sequencings_notebooks_and_scripts) and permanently archived on Zenodo (50). Briefly, the algorithm first identified reads containing the T-DNA right-arm junction sequence, allowing up to two mismatches to accommodate sequencing error and extracted the adjacent genomic flanking sequence. These flanking sequences were aligned to the *T. thermophilus* reference genome (assembly MycTherm_2019, available at the DOE JGI MycoCosm portal) (13) using BLASTn with a minimum alignment identity of 95% and an E-value threshold of 0.1. A barcode was considered to map uniquely only if the highest-scoring genome alignment exceeded the second-best alignment by at least 10 bitscore units. Barcodes differing by single-base sequencing errors were collapsed, and barcodes mapping to nearly identical positions (<10 bp apart) were resolved by retaining the most abundantly supported locus. Barcodes classified as multi-locus, ambiguous, or insert-derived were excluded. Each insertion was assigned a classification (single, multi-locus, concatameric, or ambiguous) based on mapping structure, and only single-locus insertions supported by at least one uniquely mapped read were retained for downstream mutant fitness calculations. The final pool file reported for each barcode, the chromosomal coordinate, strand orientation, gene annotation, and corresponding read abundance. The resulting pool file was then annotated by intersecting insertion coordinates with gene boundaries defined in the *T. thermophilus* genome annotation (GFF) file, thereby assigning each barcode to a specific gene (or intergenic region) and producing a final gene-level insertion index used for subsequent fitness scoring. Repetitive elements in the *T. thermophilus* reference genome were identified using RepeatMasker v4.2.2 (51) with the Dfam repeat database (52) under default parameters.

### High-throughput phenotyping and quantification of temperature-dependent fitness in the insertional mutant pool

For a given replicate of quantification of abundance of mutant across the pool of insertional mutants (Barseq), we proceeded as follows. A 1 mL frozen aliquot of a given mutant pool (Batch 1 or Batch 2; see above) was spread onto two 150 × 15 mm VMM +2% sucrose agar plates (500 µL per plate) and incubated at 37°C for 6 days in the dark to allow full conidiation. Conidia harvested from these expanded plate cultures were pooled and split into two inocula for growth: 50 mL liquid VMM + 2% sucrose was inoculated with 1 × 10⁶ conidia/mL in each case, after which one was incubated at 37°C and the other at 50°C for 24 h at 200 rpm under dark conditions. For each, mycelial biomass was harvested for genomic DNA extraction using Quick-DNA Fungal/Bacterial Miniprep Kit (Zymo Research D6005). Unique barcode regions were amplified using Q5 High-Fidelity DNA Polymerase with GC Enhancer (NEB M0491S) and primers targeting constant priming sites flanking each barcode, yielding a ~185 bp Illumina-ready amplicon (53). Amplicons were purified using Zymo DNA Clean & Concentrator (D4014), quantified using a Qubit 3.0 fluorometer, pooled in equimolar ratios within each batch. Replicates of Batch 1 (the ~4,000-mutant library) were sequenced on an Illumina MiSeq platform, and replicates of Batch 2 (the ~13,000-mutant library) were sequenced on an Illumina NextSeq P2 platform (100-cycle single-end). When necessary, libraries were size-selected using a Pippin Prep (Sage Biosciences) and spiked with 15% PhiX to increase base diversity.

Sequencing reads were processed using a modified RBseq_Count_BarCodes pipeline (50). Barcode counts were normalized by total-count scaling and linked to genomic insertion sites via the annotated pool file; gene-level abundance estimates were derived from uniquely mapped, single-locus barcodes only. As an internal quality control, genome-wide insertion density was summarized in 50-kb chromosomal bins, and replicate concordance was assessed by pairwise scatter plotting of normalized barcode abundances with coefficients of determination (*R*²) from ordinary least-squares regression. Barcodes whose insertion position from Tnseq mapped to multiple promoters or genes were eliminated from downstream analysis, as was any gene for which all insertion mutants in all replicates had fewer than five observed reads.

To test for differential abundance between temperatures of the mutants of a given gene, we proceeded as follows. We calculated the mean normalized abundance across all insertional mutants from all 37°C replicates and divided the value of the normalized abundance from each insertional mutant in each 50°C replicate by this value. Log₂ transformation of these ratios yielded per-gene fold-change estimates. For this baseline-normalized analysis, statistical testing was restricted to genes represented by ≤15 uniquely mapped barcodes, to prevent genes with very high insertion density from disproportionately influencing variance structure. For each such gene, a one-sample Wilcoxon signed-rank test was used to determine whether the median log₂(50°C/37°C) differed from 0 (i.e., ratio = 1). *P*-values were corrected using the Benjamini–Hochberg procedure, and genes were designated as temperature-responsive when the absolute value of the mean log₂(50°C/37°C) ≥ 1 and FDR < 0.05. We also computed the standard deviation and coefficient of variation (CV%) across all available replicate log₂(50°C/37°C) measurements.

### Single-gene validation of screen hits

#### Construction of single-gene deletion mutants

Single-gene knockout mutants were generated in the Δ*ku80* background of *T. thermophilus* to promote homologous recombination (54). For each target gene, we PCR-amplified the upstream and downstream genomic flanking regions, typically 500 bp to 1 kb each, depending on the locus. We assembled these flanking regions on either side of the hygromycin resistance marker (hph), which was amplified from the pLH159RB plasmid. The flanking regions and hph cassette were inserted into the pCAMBIA1302 binary vector, which had been linearized by *Xho*I and *BssH*II digestion to remove the native GFP cassette and create compatible ends for assembly. Constructs were assembled using NEBuilder HiFi DNA Assembly Master Mix (New England Biolabs, #E5520S). The assembled plasmids were transformed into *E. coli* DH5α, and transformants were selected on LB agar supplemented with 50 µg/mL kanamycin. We screened positive colonies by colony PCR, and plasmid DNA was purified using a miniprep kit (QIAprep Spin Miniprep Kit #27,106). Correct assembly was verified by diagnostic restriction enzyme digest and Sanger sequencing across the junction regions to confirm the integrity of the flanking regions and hph cassette. The complete list of plasmids generated in this study is provided in Table 2, and all primers used for cassette construction are listed in File S5.

The verified pCAMBIA1302-based deletion vectors were introduced into *A. tumefaciens* strain LBA4404 by electroporation. Transformants were recovered on low-salt LB agar supplemented with 100 μg/mL kanamycin and 50 μg/mL rifampicin to select for plasmid maintenance and the bacterial background, respectively. Gene deletion in *T. thermophilus* was then carried out by *Agrobacterium*-mediated transformation following the protocols described in previous studies (47). Briefly, freshly harvested conidia (~4 × 10⁷ per plate) were co-cultivated with *A. tumefaciens* on a cellophane membrane placed directly on IM agar plates containing 500 μM acetosyringone and incubated at 28°C for 96 h as above. After a 96-h incubation period, the cellophane membrane containing the grown co-cultivation mixture was subsequently transferred to the selection plates (VMM + 2% sucrose supplemented with 200 μg/mL hygromycin and 100 μg/mL carbenicillin) and incubated at 37°C for 4–5 days to recover primary transformants. Hygromycin-resistant colonies were then re-streaked to secondary selection plates containing the same antibiotic and incubated for an additional 10 days to promote full conidial development and ensure stable genomic integration prior to strain purification and downstream verification.

#### Molecular verification of single-gene validation mutants

To verify each gene deletion, we first grew conidiospores from the hygromycin-resistant transformants in 50 mL VMM + 2% sucrose for 72 h at 37°C and 200 rpm in the dark and extracted genomic DNA using the Quick-DNA Fungal/Bacterial Miniprep Kit (Zymo Research, D6005). We confirmed the gene deletions using a two-step PCR strategy. First, we performed 5′ and 3′ verification PCRs using primer pairs located outside the upstream and downstream flanking regions of the targeted locus. This allowed us to distinguish between the wild-type allele and the deletion allele based on product size. For each mutant, we observed a single band corresponding to the expected size of the deletion allele, which differed from the native allele band amplified from the Δ*ku80* parental strain. Second, we used internal ORF-specific primers to test for the presence or absence of the native gene. The deletion strains showed no internal ORF product, while the Δku80 parental strain produced the expected amplicon, confirming loss of the target coding region. PCRs were performed using Q5 High-Fidelity DNA Polymerase (NEB, M0491S) with GC enhancer, and products were resolved on 1% agarose gels. Strains that met both criteria—correct external PCR size and absence of the internal ORF—were purified further by single-conidium isolation to ensure clonal homogeneity. For a subset of mutants, we also sequenced the cassette–genome junctions to confirm precise integration and rule out partial or ectopic events. Primer sequences used for verification are provided in File S5.

#### Phenotypic validation of single-gene validation mutants

For the liquid growth assay of single-gene validation mutants, because strains differed in germination efficiency at 37°C and 50°C, we designed a scheme in which all strains began the hyphal-growth phase with a similar biomass input, allowing us to assess post-germination hyphal growth independently of initial germination rate. We achieved this by pre-germinating each strain and replicating in turn at 45°C and ensuring consistent growth-ready inocula in liquid culture, as follows. For a given replicate, conidia were first harvested from fully sporulated cultures, washed once in sterile water, and counted using a hemocytometer. We next used this to establish a pre-culture by inoculating 10 mL of the conidial suspension (~2 × 10^7^ conidiospores/mL) into 90 mL of VMM + 2% sucrose in a 500 mL Erlenmeyer flask, sealing with foil, and pre-germinating for 24 h at 45°C with shaking at 200 rpm under standard dark conditions. We then dispensed 5 mL pre-germinated culture (containing mycelium suspended in medium) into each of three 250 mL Erlenmeyer flasks containing 45 mL fresh VMM + 2% sucrose to establish technical replicate cultures. Replicate flasks were incubated in VMM + 2% sucrose at 37°C or 50°C for 18 h with shaking at 200 rpm under dark conditions. At the end of the incubation, mycelia were harvested by filtration through Miracloth, dried overnight at 50°C, and weighed to obtain the final dry biomass (mg). Following incubation, mycelia were collected by filtration through Miracloth, washed with sterile water, and dried overnight at 50°C to constant weight. The dry biomass (mg) was recorded for each culture. In a separate checking step for each replicate, we also took an additional 5 mL of the pre-germinated culture (apart from the inocula used for growth assays), dried it overnight at 50°C, and weighed it. The results from these checks revealed little variation between replicates or genotypes (7.0–11.0 mg), confirming that 5-mL aliquots used as inocula for growth would be consistent with respect to growth-ready biomass. Biological replicates were performed with *n* ≥ 3 per strain per condition. For visualization in heatmaps, replicate measurements were aggregated as mean values. Full replicate-level data are provided in File S4.

Germination efficiency was assessed as follows. For a given replicate, 150 µL of a conidial suspension (~2 × 10^7^ conidiospores/mL) was inoculated into 1.5 mL VMM + 2% sucrose in each well of a 24-well plate (Cellvis #P24-1.5H-N). Plates were incubated at 37°C or 50°C with shaking at 200 rpm for 10 h in the dark. To ensure uniform sampling, wells were briefly mixed by pipetting with a P1000 immediately before microscopy. This mixing step resuspended settled conidia and did not alter germination conditions. For microscopy, we withdrew 10 μL of the culture from each well and mounted it on a microscope slide for imaging. Germination was scored using a compound light microscope (National Ecoline D-ELDB Binocular Digital Microscope) at 400× magnification under brightfield illumination. A conidium was defined as germinated when the emerging germ tube exceeded the diameter of the spore body. For each replicate, ≥200 conidia were counted manually to calculate percent germination. Each strain and temperature condition was assayed in ≥ 3 biological replicates.

### Assays of zinc effects on germination

Zinc-dependent effects on germination were assessed in liquid VMM supplemented with 2% sucrose using *T. thermophilus* Δ*ku*80 or Δ*zip* strains, as indicated. Vogel’s minimal medium was prepared with graded ZnSO₄ concentrations by adjusting the trace element stock to yield final zinc levels of 0.5, 1, 3, 5, or 10 mg/L ZnSO₄ in the working 1× medium. (Standard VMM contains 5 mg/L ZnSO_4_.) For each zinc condition, 150 µL of a conidial suspension (4 × 10^7^ conidiospores/mL) was inoculated into 1.5 mL zinc-adjusted Vogel’s medium in each well of a 24-well plate (Cellvis #P24-1.5H-N). Plates were incubated at 37°C or 50°C with shaking at 200 rpm for 10 h in the dark. Wells were briefly mixed by pipetting with a P1000 immediately prior to sampling to ensure uniform suspension. For microscopy, 10 µL of culture was mounted on a glass slide and imaged using a compound light microscope at 400× magnification under brightfield illumination. A conidium was considered germinated when the germ tube length exceeded the diameter of the spore body. For each replicate, at least 200 conidia were manually counted to calculate the percent germination. Each zinc condition and temperature combination was assayed in ≥3 biological replicates. For Δ*zip* assays shown in Fig. 5B, germination was quantified at low (0.5 mg/L) and high (10 mg/L) zinc concentrations using the same conditions, incubation times, and scoring criteria as for wild-type assays.

### Statistical analysis

All data processing and statistical analyses were performed in Python (v3.10) using the pandas, numpy, scipy, statsmodels, matplotlib, and seaborn Python packages (55). Barcode abundance tables from the pooled mutant screens were normalized by total-count scaling. Gene-level fitness effects were quantified using the following two complementary statistical frameworks: (i) a Mann–Whitney U test comparing barcode abundance distributions between 37°C and 50°C, and (ii) a baseline-normalized log₂ fold-change approach in which the mean normalized abundance across 37°C replicates was used as a reference, and a one-sample Wilcoxon signed-rank test assessed whether the median log₂(50°C/37°C) differed from zero. Resulting *P*-values were corrected for multiple testing using Benjamini–Hochberg false discovery rate (FDR) control, and genes were classified as temperature-responsive when mean log₂FC ≥ 1 and FDR < 0.05. Biological replicate reproducibility was evaluated by pairwise scatter analysis of normalized barcode abundances, with coefficients of determination computed from ordinary least-squares regression. Volcano plots and coefficient-of-variation summaries were generated directly from the baseline-normalized log₂ fold-change tables.

For germination and growth phenotyping assays, temperature and genotype effects were assessed using two-way ANOVA (strain × temperature) implemented in the statsmodels Python package (55). Where a significant strain × temperature interaction was detected, *post-hoc* comparisons were conducted. For growth, Tukey’s HSD was applied to the strain factor to identify genotypes that differed from Δ*ku80* across temperatures, with significance determined by the Tukey-adjusted *P*-value. Heatmaps were generated with seaborn, and significance markers were placed directly on the row margins. All figures were generated in Python and finalized in Inkscape software version 1.4.2.

## Data Availability

Raw sequencing data are available from the NCBI Sequence Read Archive (SRA) under accession number PRJNA1355313. Germination assay micrographs are available on Figshare at https://doi.org/10.6084/m9.figshare.30593345. T-DNA sequencing data are in File S1, and barcode sequencing data are in File S3. Growth, conidiation, and germination data for wild-type and single-gene validation mutants are in File S4. Plasmids generated in this study are listed in Table 2, and all primers used for cassette construction and strain verification are provided in File S5.

## SUPPLEMENTAL MATERIAL

File S1 mbio.03907-25-s0001.xlsx T-DNA junction sequencing of *T. thermophilus* insertional mutants.

File S2 mbio.03907-25-s0002.xlsx Genome-wide RepeatMasker annotations for the *T. thermophilus* reference genome.

File S3 mbio.03907-25-s0003.xlsx Genome-wide BarSeq fitness profiles of *T. thermophilus* at 37°C and 50°C.

File S4 mbio.03907-25-s0004.xlsx Raw measurements of conidiation, biomass accumulation, and germination of wild-type and single-gene validation mutant *T. thermophilus*.

File S5 mbio.03907-25-s0005.xlsx Primer sequences used for construction of deletion cassettes and validation of gene deletions by PCR.

Figure S1 mbio.03907-25-s0006.pdf Reproducibility of barcode abundance measurements across biological replicates.

Figure S2 mbio.03907-25-s0007.pdf PCR confirmation of targeted gene deletions in *T. thermophilus*.

Supplemental legends mbio.03907-25-s0008.docx Descriptive legends for supplemental material.

ASM does not own the copyrights to Supplemental Material that may be linked to, or accessed through, an article. The authors have granted ASM a non-exclusive, world-wide license to publish the Supplemental Material files. Please contact the corresponding author directly for reuse.
