# Supplementary figures and images for: High-throughput screening reveals mechanisms of environmental control of germination in a fungal thermophile
Source: mBio. 2026 Apr 20;17(5):e03907-25. doi: 10.1128/mbio.03907-25 (PMC13170357; doi:10.1128/mbio.03907-25)

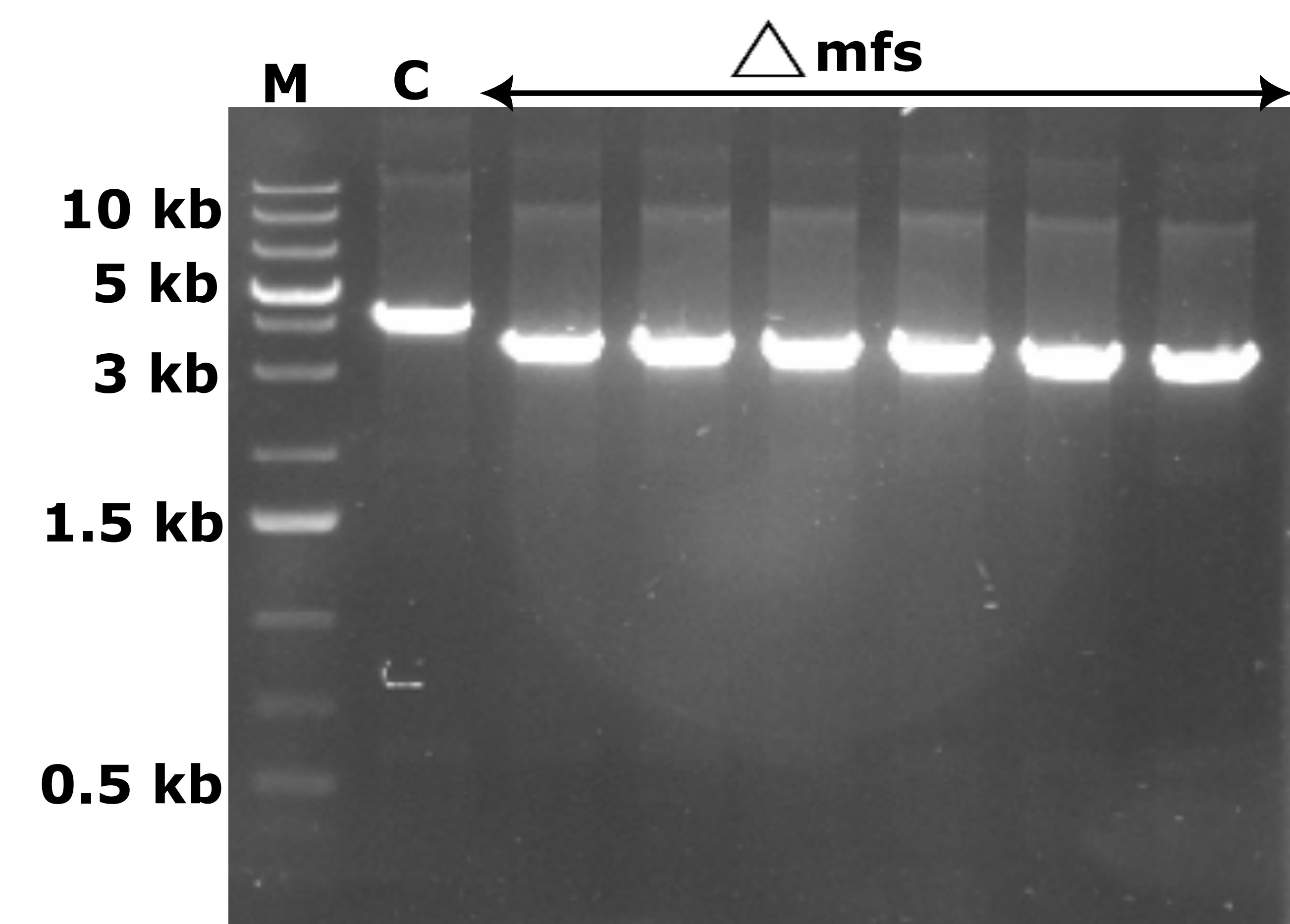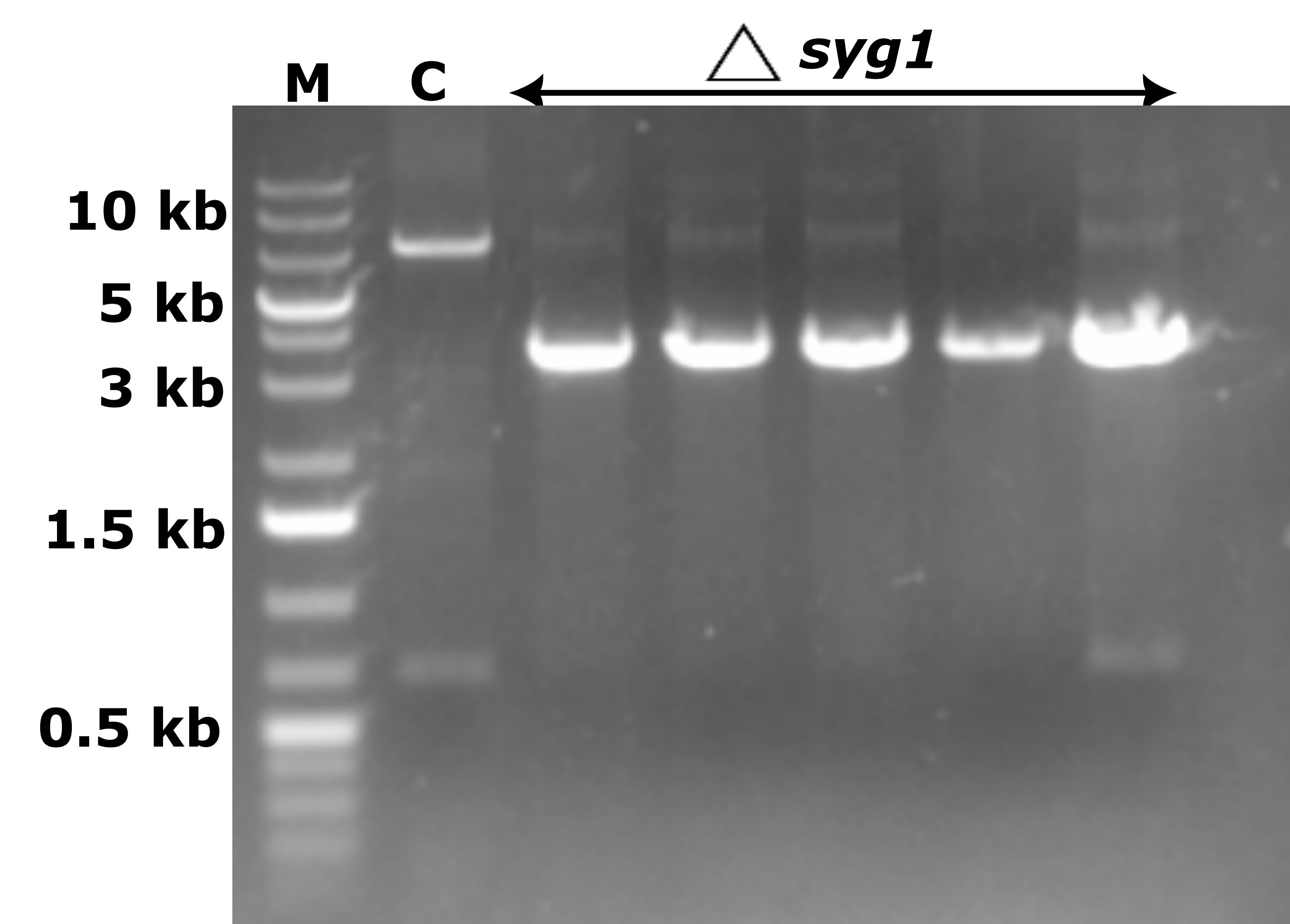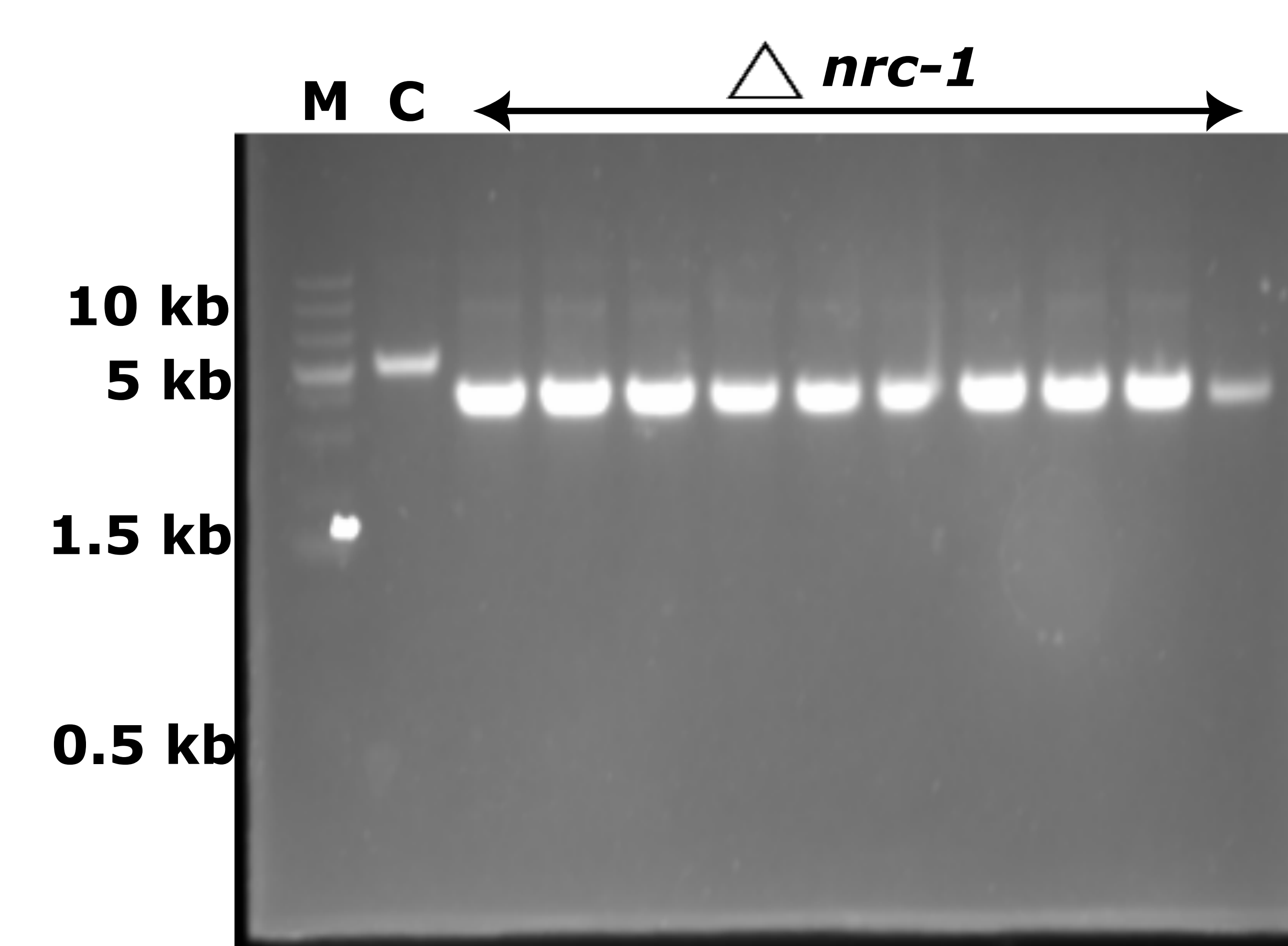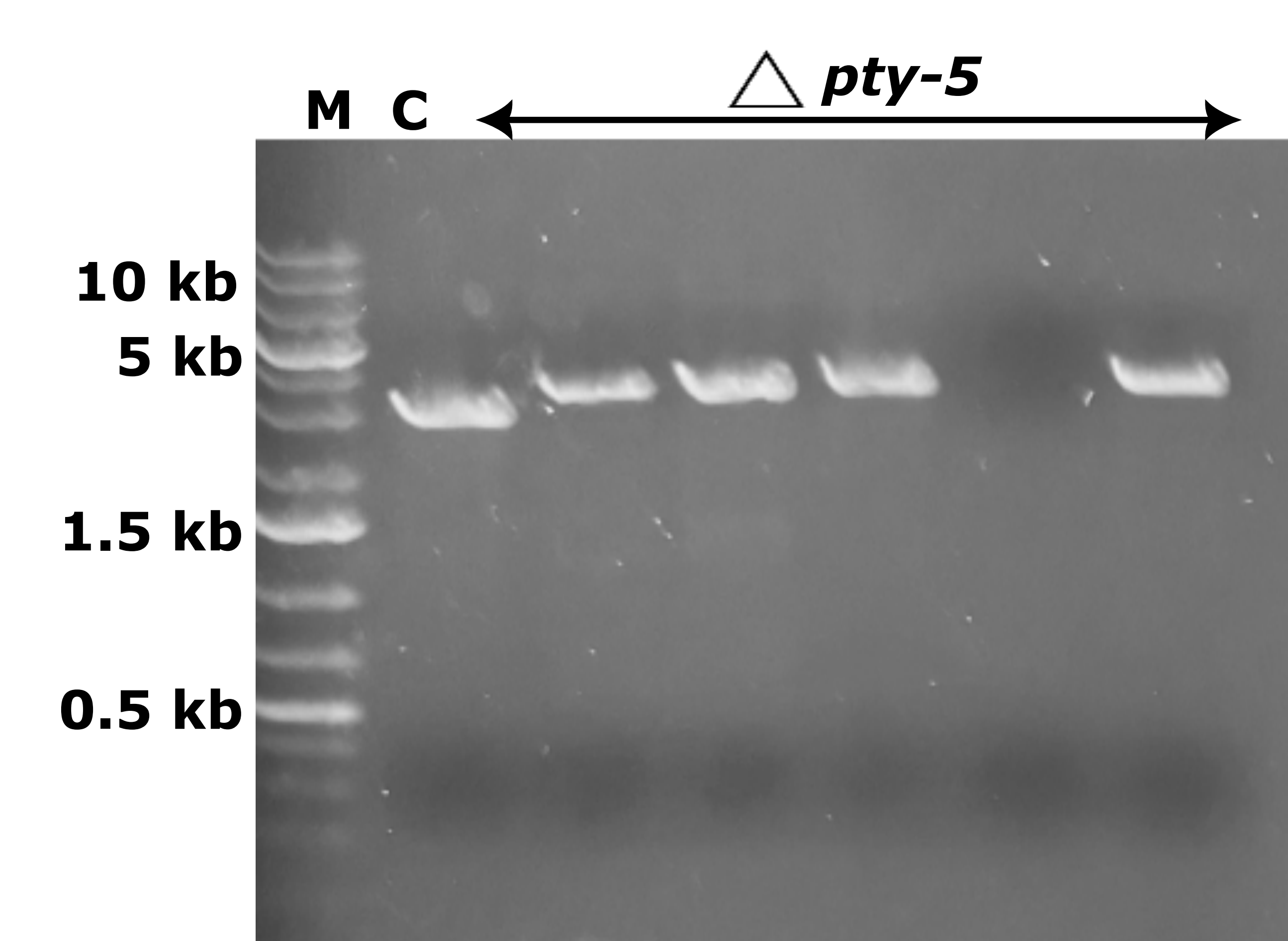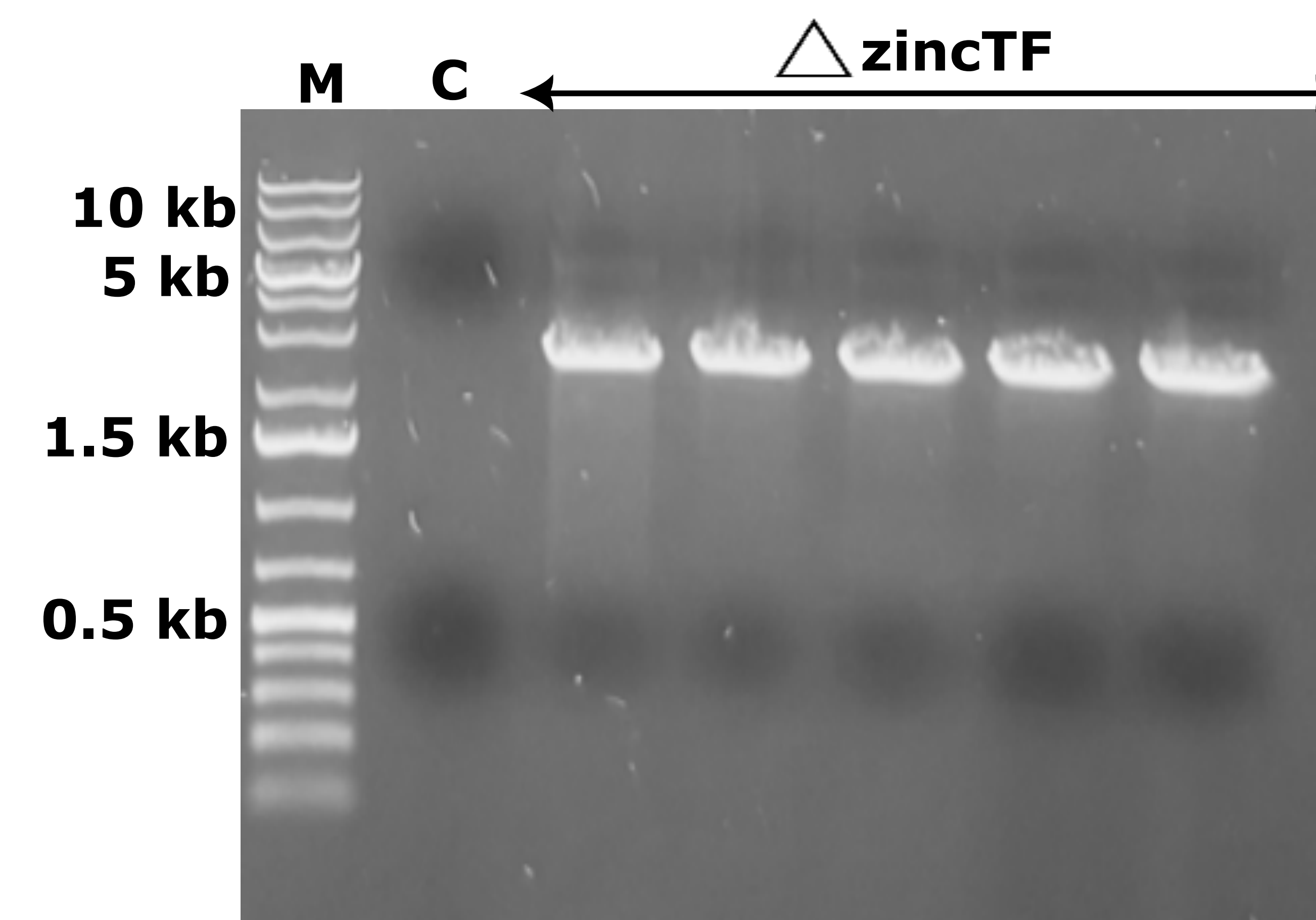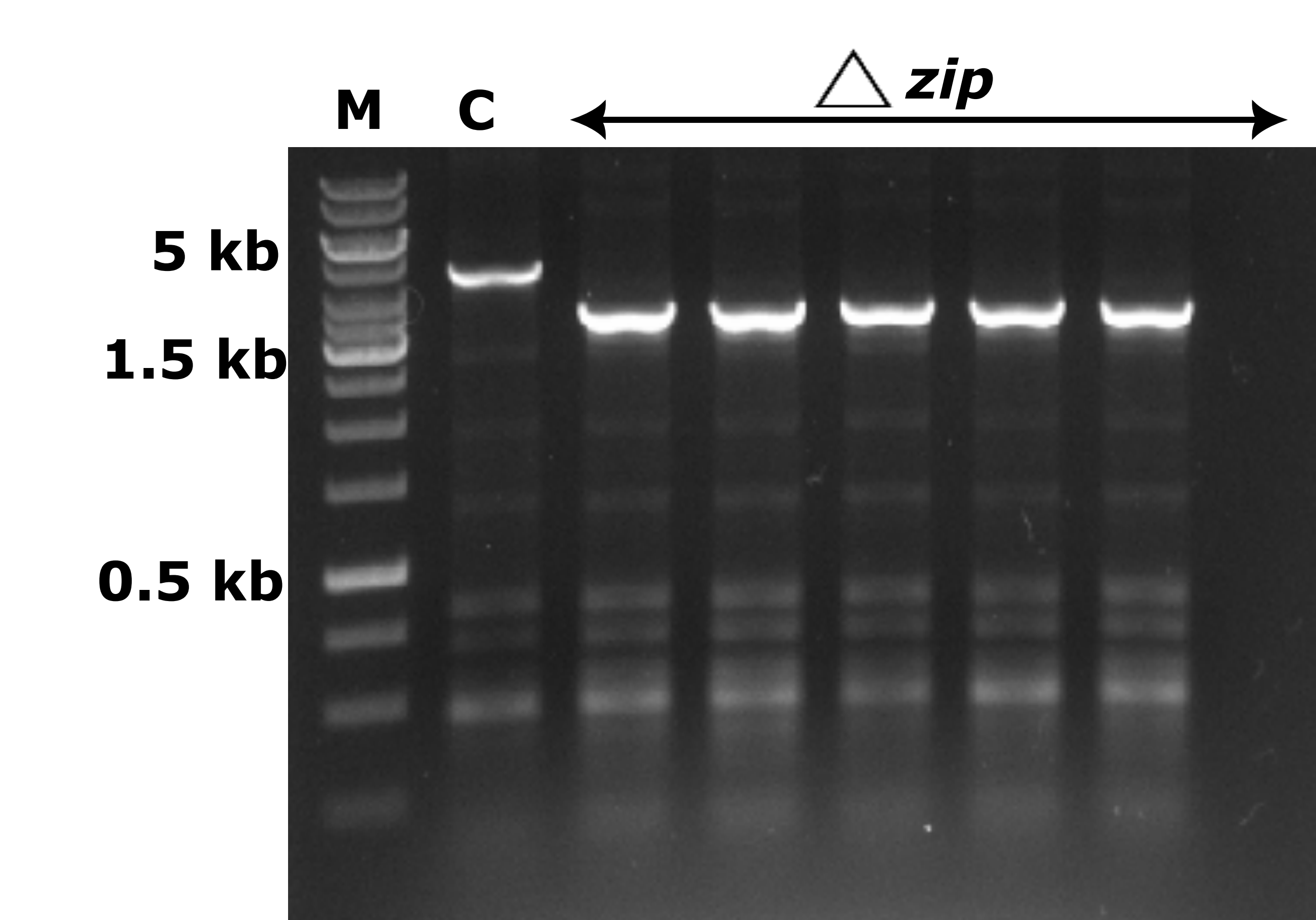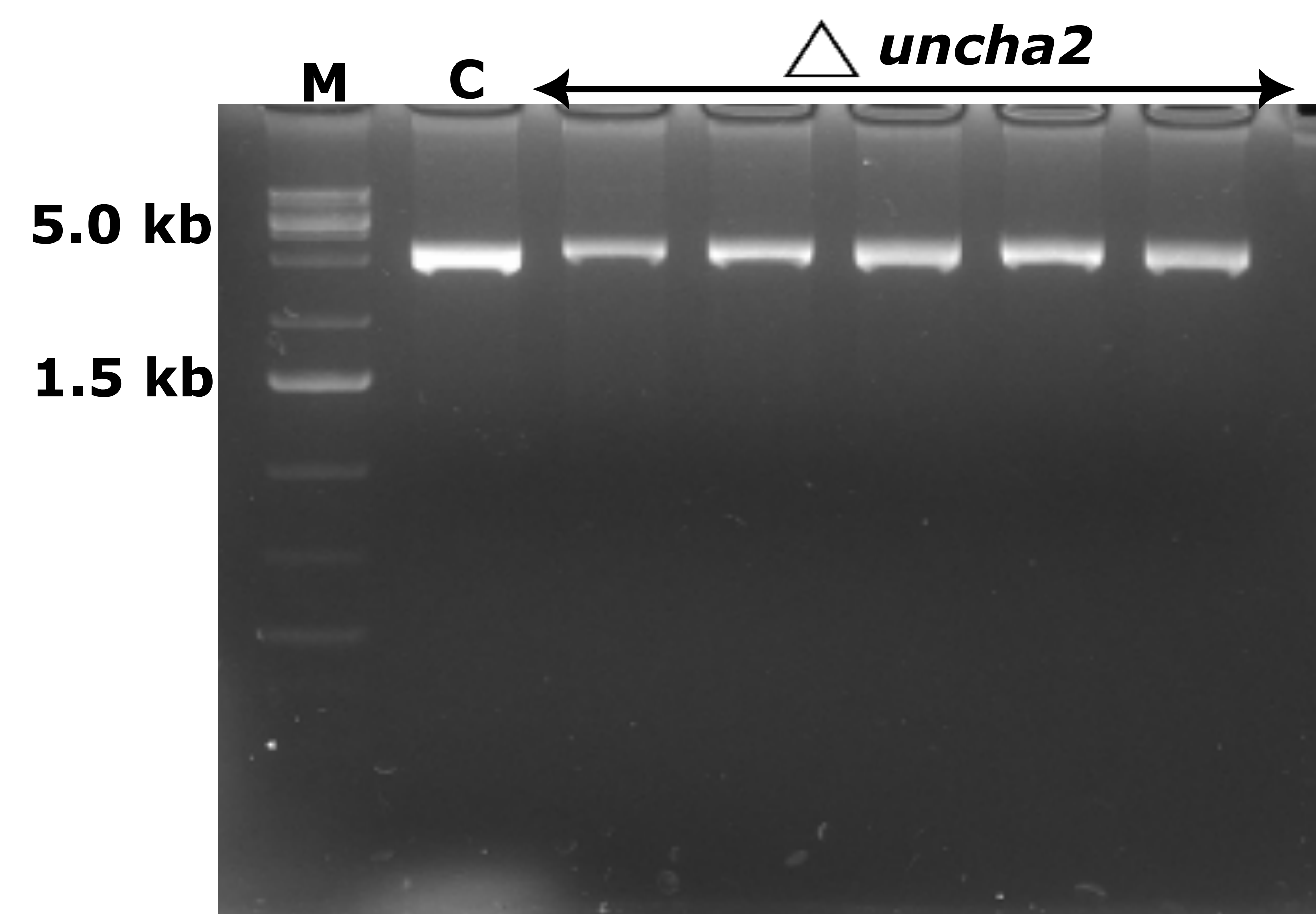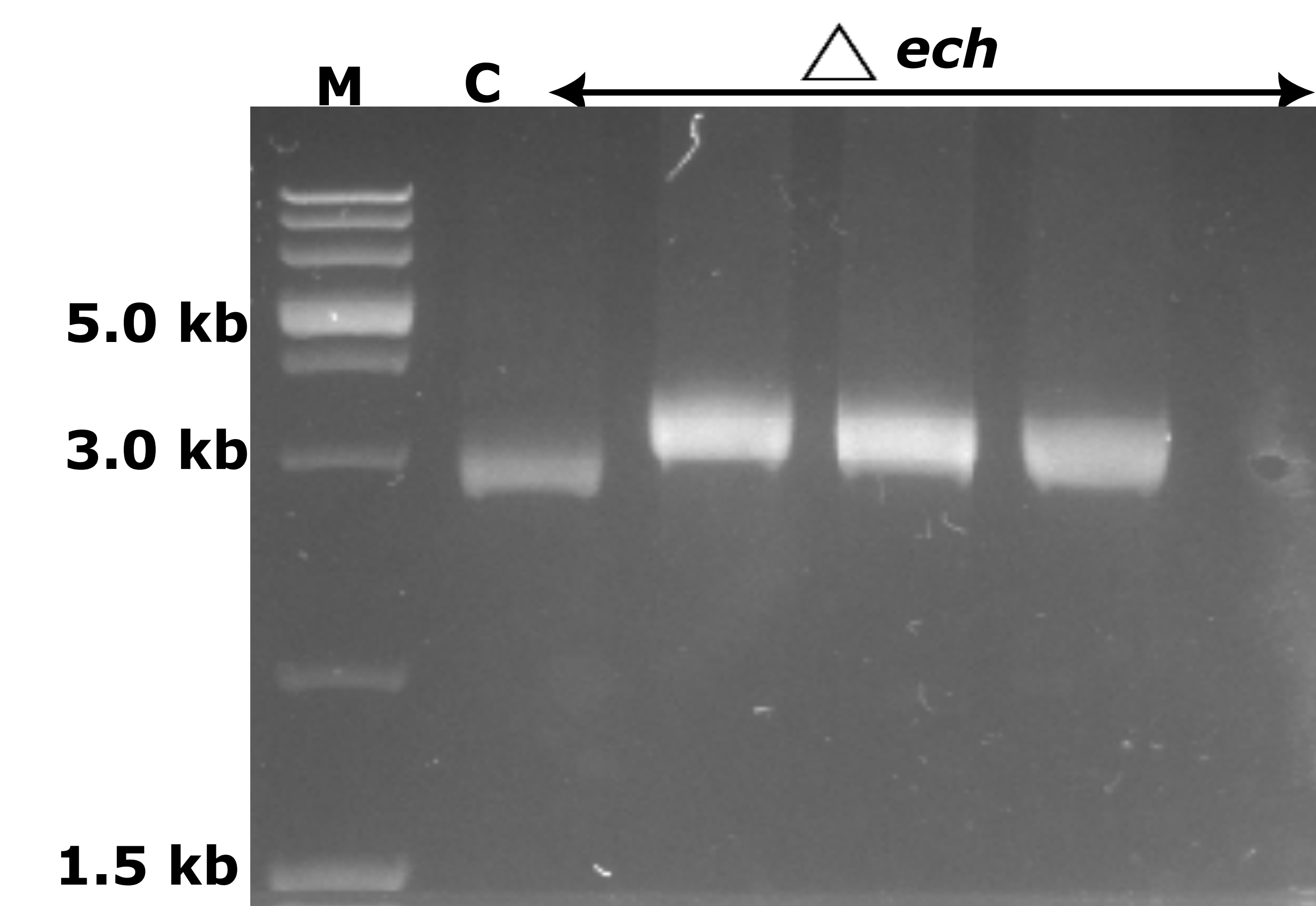

Supplement: Figure S2 — PCR confirmation of targeted gene deletions in T. thermophilus. [file mbio.03907-25-s0007.pdf]
