# Supplementary material for: High-throughput screening reveals mechanisms of environmental control of germination in a fungal thermophile
Source: mBio. 2026 Apr 20;17(5):e03907-25. doi: 10.1128/mbio.03907-25 (PMC13170357; doi:10.1128/mbio.03907-25)
Supplement: Supplemental legends — Descriptive legends for supplemental material. [file mbio.03907-25-s0008.docx]

Supplementary figure captions

**Supplementary Figure 1. Reproducibility of barcode abundance measurements across biological replicates.** Data are as in Figure 3B of the main text, except that panels A–C correspond to replicate comparisons at 37°C for the Batch 1 mutant pool (sets A1–A2, B1–B2, and C1–C2); D–E show replicate comparisons at 50°C for the Batch 1 mutant pool (sets A1–A2 and B1–B2); F-G correspond to replicate comparisons at 37°C for the Batch 2 mutant pool; H-I correspond to replicate comparisons at 50°C for the Batch 2 mutant pool. The coefficient of determination (R²) for each comparison is shown in the top left of each plot.

**Supplementary Figure 2. PCR confirmation of targeted gene deletions in *T. thermophilus*.** Diagnostic PCR was used to verify homologous replacement of each target locus in the deletion strains (Δ*mfs*, Δ*syg1*, Δ*nrc-1*, Δ*pty-5*, Δ*zincTF*, Δ*zip*, *ΔMYCTH_2313252* and Δ*ech*). For each gene, lanes labeled C correspond to the parental Δ*ku80* control, and adjacent lanes show amplicons from independent deletion-mutant isolates. Loss of the wild-type band and/or the presence of the expected deletion-specific product confirms successful gene replacement at the target locus. M refers to the DNA size marker. The expected size bands for each of the wild type and transformant PCRs are listed in Supplementary File 4.

Supplementary file captions

**Supplementary File S1. T-DNA junction sequencing of *T. thermophilus* insertional mutants.** Listed are all mapped insertion mutants in the *T. thermophilus* genome. Columns report barcode and reverse-complement sequences (barcode, rcbarcode), total read support (nTot) and primary-locus support (n), genomic location (scaffold, strand, pos), mapping classification (type), read support for the main mapped site (nMainLocation) and for T-DNA border–derived reads (nInsert), all alternate genomic alignments (All genomic mappings), and annotated overlapping or nearest gene (gene).

**Supplementary File S2. Genome-wide RepeatMasker annotations for the *T. thermophilus* reference genome.** This file contains RepeatMasker v4.2.2 annotations generated using the Dfam repeat database. Columns report chromosome, start coordinate, end coordinate, repeat feature classification, motif annotation, score, strand, and full RepeatMasker attribute string.

**Supplementary File S3. Genome-wide BarSeq fitness profiles of *T. thermophilus* at 37^°^C and 50^°^C.** This file contains three worksheets reporting results of gene-level tests for differential abundance between temperatures from BarSeq data. In each sheet, GeneID is the annotated gene identifier; Mean_log2FC is the mean log₂ fold change in mutant abundance (50°C/37°C); P-Value is the nominal *p*-value from the Wilcoxon rank-sum test for differential abundance between temperatures; Adjusted_P-Value is the multiple-testing–corrected *p*-value (Benjamini–Hochberg); protein_names is the predicted or annotated protein name; Std_log2FC is the standard deviation of mean log₂ fold change in mutant abundance (50°C/37°C) across mutants and replicates; and CV_log2FC_percent is the coefficient of variation of mean log₂ fold change in mutant abundance (50°C/37°C) across mutants and replicates, expressed as a percentage. Worksheet 1 includes results from all genes with BarSeq data that met our quality control threshold (≤15 detected insertions; see Methods). Worksheet 2 reports genes whose mutants exhibited temperature-responsive fitness that met significance and effect-size thresholds for which mean log₂ fold change in mutant abundance (50°C/37°C) ≥ 1. Worksheet 3 reports genes that met significance and effect-size thresholds for which mean log₂ fold change in mutant abundance (50°C/37°C) ≤ –1.

**Supplementary File S4. Raw measurements of conidiation, biomass accumulation, and germination of wild-type and single-gene validation mutant *T. thermophilus*.** This file contains five worksheets reporting unprocessed measurements underlying Figures 1, 4, and 5. Worksheet 1 reports raw counts of conidia harvested from wild-type cultures grown on solid VMM + 2% sucrose at 37°C, 45°C, and 50°C. Columns report temperature and conidia count. Worksheet 2 reports dry-weight biomass measurements for wild-type cultures grown in liquid VMM containing 2% sucrose at 30°C, 37°C, 45°C, 50°C, and 55°C. Columns report temperature and growth (mg). Worksheet 3 reports Biomass accumulation dry-weight biomass measurements for all deletion mutants and the Δ*ku*80 parent grown in liquid VMM + 2% sucrose at 37°C and 50°C. Columns report strain, temperature, and growth (mg). Worksheet 4 reports microscopy-based germination measurements for all deletion mutants and the Δku80 parent at 37°C and 50°C. Columns report strain, temperature, total number of conidia, number of germinated conidia, and percentage germination. Worksheet 5 reports the raw germination data for Δku80 conidia exposed to varying Zn concentrations at 37°C and 50°C. Columns report zinc concentration, temperature, total number of conidia, number of germinated conidia, and percentage germination.

**Supplementary File S5. Primer sequences used for construction of deletion cassettes and validation of gene deletions by PCR.** This file contains three worksheets detailing the oligonucleotides used in this study for TnSeq/BarSeq library preparation, deletion-cassette construction, and PCR-based mutant verification. Worksheet 1 reports the primers used for Tnseq and Barseq amplifications, indexing, and nested enrichment of T-DNA junction fragments. The columns report primer_name, primer_sequence, index_sequence (for multiplexing), and index_reverse (custom P1 index primer). Worksheet 2 reports the primers used to assemble deletion cassettes and to verify correct integration in transformants by PCR. Columns report GeneID, primer_name, primer_sequence, and purpose (e.g., 5′ flank amplification, junction PCR, confirmation PCR). Worksheet 3 reports the expected amplicon sizes for wild-type (Δku80 background) and deletion mutants after cassette integration. Columns report gene, primer_set, expected_size_in_Δku80 (kb), and expected_size_in_mutant (kb).
