# Supplementary material for: High-throughput screening reveals mechanisms of environmental control of germination in a fungal thermophile
Source: mBio. 2026 Apr 20;17(5):e03907-25. doi: 10.1128/mbio.03907-25 (PMC13170357; doi:10.1128/mbio.03907-25)
Supplement: Figure S1 — Reproducibility of barcode abundance measurements across biological replicates. [file mbio.03907-25-s0006.pdf]

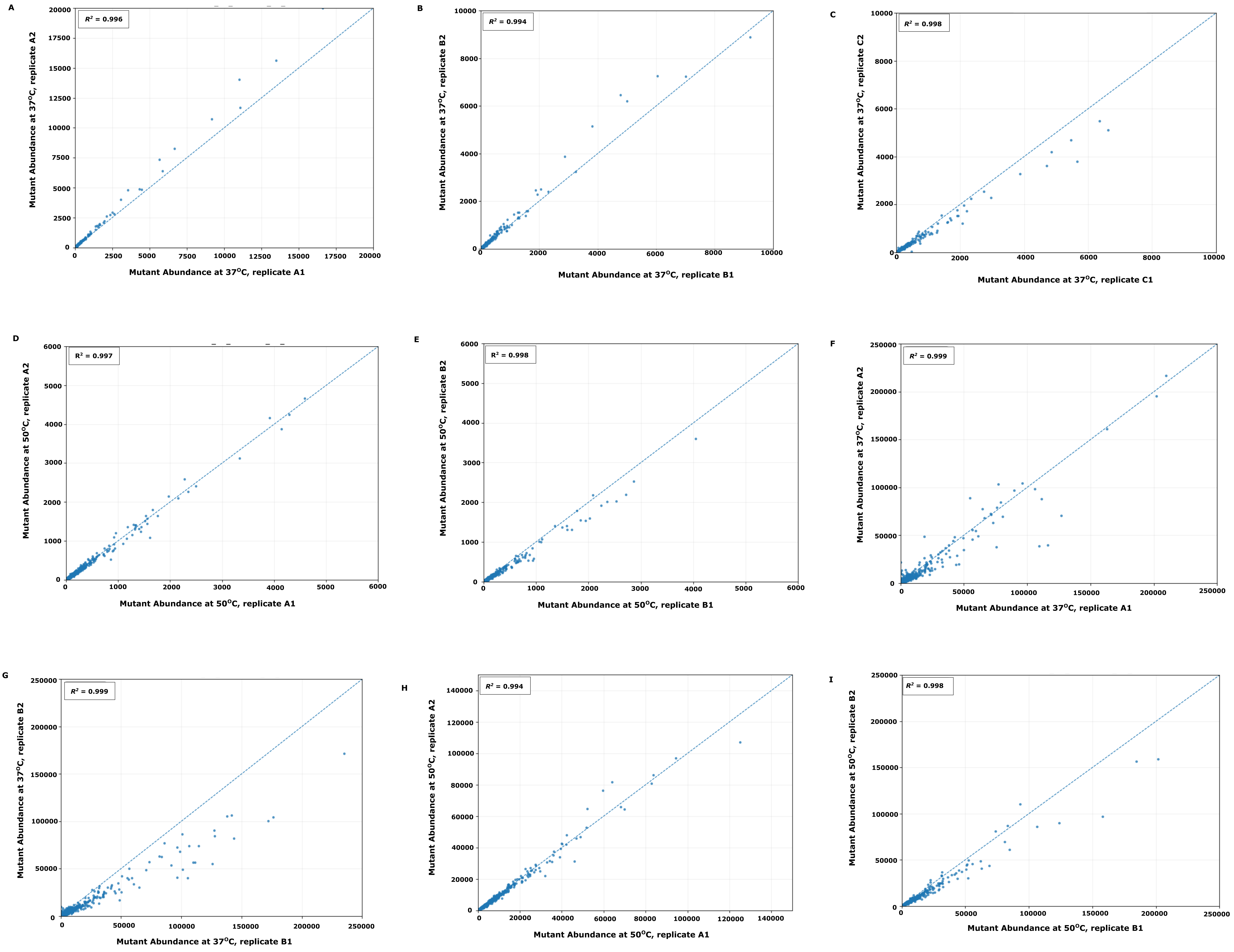

A
R² = 0.996
Mutant Abundance at 37°C, replicate A2
Mutant Abundance at 37°C, replicate A1
B
R² = 0.994
Mutant Abundance at 37°C, replicate B2
Mutant Abundance at 37°C, replicate B1
C
R² = 0.998
Mutant Abundance at 37°C, replicate C2
Mutant Abundance at 37°C, replicate C1
D
R² = 0.997
Mutant Abundance at 50°C, replicate A2
Mutant Abundance at 50°C, replicate A1
E
R² = 0.998
Mutant Abundance at 50°C, replicate B2
Mutant Abundance at 50°C, replicate B1
F
R² = 0.999
Mutant Abundance at 37°C, replicate A2
Mutant Abundance at 37°C, replicate A1
G
R² = 0.999
Mutant Abundance at 37°C, replicate B2
Mutant Abundance at 37°C, replicate B1
H
R² = 0.994
Mutant Abundance at 50°C, replicate A2
Mutant Abundance at 50°C, replicate A1
I
R² = 0.998
Mutant Abundance at 50°C, replicate B2
Mutant Abundance at 50°C, replicate B1
